# A novel clinically relevant antagonistic interplay between prolactin and oncogenic YAP-CCN2 pathways as a differentiation therapeutic target in breast cancer

**DOI:** 10.1038/s41419-025-07547-7

**Published:** 2025-03-29

**Authors:** Xueqing Liu, Alaa Moamer, Roger Gomes da Silva, Aidan Shoham-Amizlev, Dana Hamam, Anwar Shams, Jean-Jacques Lebrun, Suhad Ali

**Affiliations:** 1 Department of Medicine, Cancer Research Program, Centre for Translational Biology, McGill University Health Centre, McGill University, Montreal, QC Canada; 2 Department of Pharmacology, College of Medicine, Taif University, Taif, Saudi Arabia

**Keywords:** Breast cancer, Targeted therapies

## Abstract

Cellular differentiation limits cellular plasticity allowing cells to attain their specialized functional characteristics and phenotypes, whereas loss of differentiation is a hallmark of cancer. Thus, characterizing mechanisms underlying differentiation is key to discover new cancer therapeutics. We report a novel functional antagonistic relationship between the prolactin (PRL)/prolactin receptor (PRLR) differentiation pathway and YAP-CCN2 oncogenic pathway in normal mammary epithelial cells and breast cancer cells that is essential for establishing/maintaining acinar morphogenesis, cell-cell junctions and the intracellular localization of apical-basal polarity protein complexes (Par, Crumb and Scrib). Importantly, using CRISPR knockout of the PRLR in MCF7, HR+ breast cancer cells, further revealed that the negative relationship between PRL/PRLR pathway and YAP-CCN2 pathway is critical in suppressing luminal-to-basal stem-like lineage plasticity. Furthermore, the clinical relevance of this interplay was evaluated using bioinformatics approaches on several human datasets, including samples from normal breast epithelium, breast cancer, and 33 other cancer types. This analysis revealed a positive correlation between PRLR and the YAP suppressor Hippo pathway and a co-expression gene network driving favourable patients’ survival outcomes in breast cancer. The therapeutic potential of this interplay was also evaluated in vitro using MDA-MB-231 cells, a preclinical model of human triple-negative breast cancer, where treatment with PRL and Verteporfin, an FDA-approved pharmacological YAP-inhibitor, alone or their combination suppressed the expression of the mesenchymal marker vimentin and the stem cell marker CD44 as well as reduced their Ki67 proliferative marker expression. Collectively, our results emphasize the pro-differentiation role of PRL/PRLR pathway in mammary and breast cancer cells and highlight that promoting PRL/PRLR signaling while inhibiting the YAP-CCN2 oncogenic pathway can be exploited as a differentiation-based combination therapeutic strategy in breast cancer.

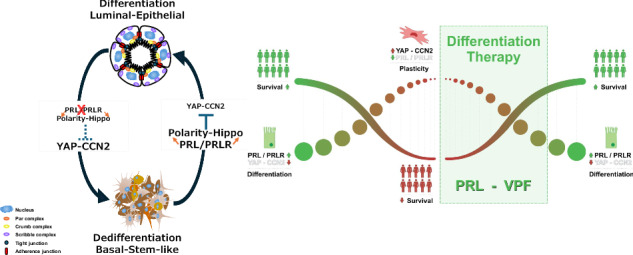

## Introduction

Recent advances in our understanding of tumorigenesis accentuate the role of tumor differentiation state and lineage plasticity along with the enrichment of stem-like cell states, as important tumor parameters driving tumor heterogeneity, cancer progression and resistance to therapy [1–4]. In response to these challenges, there is growing interest in characterizing mechanisms of cellular reprogramming, restoring forward-differentiation of tumor cells as potential therapeutic strategies targeting tumor plasticity. These approaches aim to limit the proliferative, stemness, epithelial-mesenchymal-transition (EMT) and metastatic capacities of tumor cells and/or rescue drug sensitivity, leading to the cessation of the aggressive tumor phenotype allowing patients to experience long-term remission [5–8]. In accordance, breast cancer is a heterogeneous disease with various molecular subtypes each displaying distinct differentiation characteristics, such as disease progression, treatment responses, relapse rates, and survival outcomes [9]. Clinically, hormone receptor-positive (HR + )/luminal A breast tumors are most differentiated with better survival outcomes, whereas the luminal B, HER2-enriched (HER2-E) and triple-negative (TNBC) subtypes show aberrant/blocked differentiation, enrichment with stem-like cells, higher proliferation and metastatic rates, and the worst survival outcomes [10, 11]. This raises the notion that therapeutic approaches based on restoring/engaging forward differentiation may also be effective treatment options for breast cancer. However, despite the advancement of breast cancer therapeutics, including anti-hormonal therapy tamoxifen, tyrosine kinase inhibitors and the recent development of novel selective estrogen receptor degraders (SERDs), cell cycle CDK4/6 inhibitors, PARP-inhibitors, immunotherapy, antibody–drug conjugates (ADCs) and inhibitors of the PI3K/protein kinase B(AKT)/mammalian target of rapamycin (mTOR), none of these approaches are differentiation-based modalities [12–14].

Differentiation of epithelial tissues is linked to the establishment/maintenance of apical-basal (A/B) polarity program, which is critical for normal cell functions, whereas deregulation of this mechanism contributes to tumorigenesis [15, 16]. Cellular asymmetry is regulated by functional polarity protein complexes that exhibit differential intracellular distribution across different membrane domains, some of which are mutually exclusive. The Crumb (Crb3, PATJ, PALS1) and Par (Par3, Par6, PKCζ) complexes are localized apically, whereas the Scribble (Scrib, Llgl, Dlg) complex shows basolateral localization. Together, these protein complexes facilitate the formation of the apicobasal axis and the assembly and maintenance of epithelial cell-cell junctions [16, 17]. Moreover, polarity proteins are known as key regulators of the tumor suppressor Hippo pathway [18–20]. The Hippo pathway consists of the activation of a set of Ser/Thr kinases, such as mammalian STE20-like protein kinase 1/2 (MST1/2) and large tumor suppressor 1/2 kinases (LATS1/2). These kinases, in turn, phosphorylate and inhibit the transcriptional coactivators Yes-associated protein (YAP) and PDZ-binding motif (TAZ), preventing their nuclear translocation and thereby suppressing their transcriptional activity [21, 22]. In solid tumors, an inactive Hippo signaling pathway allows YAP/TAZ nuclear accumulation and interaction with the transcriptional enhanced associate domain (TEAD) protein family of transcription factors, driving an oncogenic transcriptional program [23]. An important downstream target of YAP and TEAD is the cellular communication factor 2 (CCN2, also known as CTGF), a member of the CCN family (CCN1-CCN6) [24]. These CCN proteins play crucial roles in development, wound healing, fibrosis, and cancer [25, 26]. Indeed, CCN2 has been reported to promote tumorigenesis in many cancer types, including pancreatic cancer, prostate cancer, lung cancer, breast cancer [27–29] among others [30, 31]. Moreover, the expression of CCN2 correlates with high tumor grade and metastasis including bone metastasis [32–35]. Therefore, both YAP and CCN2 are considered therapeutic targets in cancer and their inhibitors are being evaluated preclinically as well as clinically in different cancer and disease conditions [36–39].

In the mammary gland, maintaining proper A/B polarity is integral to the alveologenesis/acinar morphogenesis and it is critical in supporting lactation by allowing the directional, intraluminal secretion of milk components [40, 41]. The hormone prolactin (PRL) is known to play an indispensable role in mammary gland lobuloalveolar development and remodeling, A/B polarity and the terminal differentiation of the mammary epithelial cells (MECs) [41–43]. The role of PRL in breast cancer is still to be fully characterized [44]. Importantly, in line with the differentiation role of PRL in the breast, PRL mRNA levels were found to be associated with more differentiated tumors, early stage, smaller tumor size, absence of distant metastasis and prolonged relapse-free survival (RFS) [45–47]. Similarly, PRLR gene expression is highest in differentiated HR+/luminal A breast cancer subtype and least expressed in the triple negative, basal-like subtype and its expression correlates with favorable prognosis [48]. Moreover, PRL/PRLR signaling was found to suppress stemness and tumorigenesis in cell models of HER2-E and TNBC [46, 49, 50]. Additionally, direct CRISPR/Cas9 knockout of the PRLR in HR+ and HER2-E breast cancer cells caused dedifferentiation/aberrant differentiation, loss of linage specificity and led to enhanced tumorigenic and metastatic phenotypes [51]. Despite this strong evidence, the interplay between PRL signaling, A/B polarity proteins, and the Hippo-YAP-CCN2 pathway in mammary and breast cancer is still to be established. Understanding these remarkable interactions holds promise for developing innovative therapeutic strategies for breast cancer.

This study delves into the regulation of A/B polarity protein complexes by PRL, shedding light on its pro-differentiation effects in the breast. Using 3D-culture models of primary mammary epithelial cells, we found that PRL signaling coordinates acinar morphogenesis and regulates the intracellular distributions of polarity protein complexes (Crumb, Par, Scribble). Notably, PRL also promoted distinct morphological features in breast cancer cellular models, with acinar-like structures in HR+ breast cancer cells and induced epithelial-like features in claudin-low mesenchymal TNBC cells. RNA-Seq data analysis of CRISPR/Cas9 knockout of the PRLR in HR+ breast cancer cells revealed the oncogenic CCN2 gene as the most upregulated target gene, highlighting an antagonistic relationship between PRLR and CCN2 gene expression. Mechanistically, PRL treatment of mammary and breast cancer cells resulted in Hippo pathway activation, thereby induced nuclear exclusion of the oncoprotein YAP. In contrast, interference with PRLR or downstream signaling JAK2 kinase led to nuclear accumulation of YAP and increased CCN2 expression. Finally, targeting the YAP-CCN2 pathway with the pharmacological inhibitor Verteporfin (VPF) restored PRL/PRLR induced-differentiation features in breast cancer cells and re-established proper intercellular junctional adherence, promoted luminal differentiation and suppressed stem-cell marker expression. Clinically, this newly discovered antagonistic relationship between PRL/PRLR and YAP-CCN2 pathways was further explored and extended to large gene expression datasets of normal and breast cancer cases as well as 33 other cancer types allowing the discovery of a PRLR/Hippo pathway gene co-expression network of favorable prognosis in breast cancer. We further employed a preclinical model of breast cancer and defined PRL and VPF by promoting differentiation and effectively suppressing tumor cell growth as a potential combination therapeutic approach in breast cancer. Overall, our results highlight that promoting PRL/PRLR signaling while inhibiting the YAP-CCN2 oncogenic pathway as a differentiation-based therapeutic opportunity in breast cancer with potential merit in other cancers. Furthermore, this differentiation-based therapeutic strategy could act synergistically with existing clinical trial treatments, potentially enhancing their efficacy and offering a more comprehensive approach to combating cancer.

## Materials and methods

### Ethical approvals

All experimental animal work was performed in a specific pathogen-free animal facility according to the guidelines and ethical regulations of the Research Institute McGill University Health Centre approved animal used protocol (#2014-7492) in accordance with Canadian Council of animal care guidelines.

### Animal and cells

Virgin C57BL/6 (Jackson Mice, 12 weeks old) female mice were used to prepare MECs following the protocol from STEMCELL Technologies Inc. (Document # 29179). Upon euthanasia, mammary glands were resected from C57BL/6 mice and transferred to a sterile glass petri dish. Mince with scalpels in a crosswise pattern until glands are rendered to a paste. Then, transfer the mammary tissue to the tube containing the 1-part Collagenase/Hyaluronidase dissociation solution (STEMCELL, 07912) with 9 parts Complete EpiCult™-B Medium (STEMCELL, 05612 was added to 05611)) supplemented with 5% fetal bovine serum (FBS, Gibco, 12483020). And then, incubate the tubes at 37 °C with shaking speed 250 rpm/min for 1.5 h. After dissociation, centrifuge the cells at 350 × *g* for 5 min and discard the supernatant. Resuspend the pellet with a 1:4 mixture of cold Hanks’ Balanced Salt Solution Modified (STEMCELL, 37150) supplemented with 2% FBS and Ammonium Chloride Solution (STEMCELL, 07800) and centrifuge at 350 × *g* for 5 min. The resultant pellet contains epithelial cell organoids as well as stromal cells and lymphocytes. Add 2 mL of pre-warmed Trypsin-EDTA (Wisent, 325-043-EL) to the partially dissociated tissue and mix by pipetting. Gently pipette up and down with a P1000 micropipettor for 1–3 min. Add 10 mL of cold Hanks’ Balanced Salt Solution Modified (STEMCELL, 37150) supplemented with 2% FBS (referred to as HF) and centrifuge at 350 × *g* for 5 min. Remove as much of the supernatant as possible. Add 2 mL of pre-warmed Dispase (5 U/mL, STEMCELL, 07913) and 200 µL of DNase I Solution (1 mg/mL, STEMCELL, 07900). Pipette the sample for 1 min with a P1000 micropipettor to further dissociate cell clumps. The sample should now be cloudy, but not stringy. Dilute the cell suspension with an additional 10 mL of cold HF and filter the cell suspension through a 40 µm Cell Strainer (STEMCELL, 27305) into a new 50 mL centrifuge tube. Centrifuge at 350 × *g* for 5 min and discard the supernatant. Seed mouse mammary cells into tissue culture flasks at a density of 2–4 × 10^3^ cells/cm^2^ in complete EpiCult™-B Medium supplemented with 10 ng/mL recombinant mouse EGF (Sigma, SRP3196), 10 ng/mL recombinant human basic Fibroblast Growth Factor (rh bFGF; STEMCELL, 02634) and 4 μg/mL (0.0004%) Heparin (STEMCELL, 07980) and 5% FBS. After 24 h, change the culture medium to serum-free complete EpiCult™-B Medium containing cytokines. Human cell lines MCF7 (HTB-22) and MDA-MB-231 (HTB-26) were obtained from ATCC and cultured in high glucose-containing Dulbecco’s modified Eagle’s medium (DMEM, wisent, 319-005-CL) supplemented with 10% FBS and 1% penicillin/streptomycin (Wisent, 450-201-EL). MCF7/SCR and MCF7/PRLR-KO cells generated by previously [51] were cultured under puromycin (0.5 μg/mL, Wisent, 450-162-XL) selection. All cells were grown at 37 °C in a humidified atmosphere with 5% CO_2_. MECs were treated with or without 250 ng/mL ovine PRL (Sigma, L6520-SIGMA), and all the human cells were treated with 250 ng/mL recombinant human PRL (rhPRL, Abcam, ab269220). MCF7/WT and MCF7/PRLR-KO cells were treated with or without 0.1 μM VPF (Selleckchem, S1786) for 48 h. All hormones and pharmacological compounds were suspended and stored according to the manufacturer’s guidelines.

### Three-dimensional (3D) culture and treatment of primary MECs

The 8-Chamber Cell Culture Slide (Celltreat, 229168) was utilized for 3D culture. Briefly, each well of the culture slide was first coated with 100 μL growth factor reduced Matrigel (Corning, CLS354234), after polymerization, 4000 cells in 100 μL growth medium were plated and allowed 1.5 h for cells to attach. 100 μL growth media containing 10% Matrigel was added on top, creating a final concentration of 5% Matrigel in full growth medium. Cells were maintained in growth medium with 5% Matrigel for 2 d for mammosphere outgrowth. The morphology of the mammospheres was evaluated after 3 d of different treatments: (1) control (CTL): 2% FBS, (2) HI: 1 μM hydrocortisone (Sigma, H4001), 5 μg/mL insulin (Sigma, I9278), and 2% FBS, (3) PRL: 2 μg/mL ovine PRL and 2% FBS, (4) HIP: 1 μM hydrocortisone, 5 μg/mL insulin, 2 μg/mL ovinePRL and 2% FBS, (5) EGF: 10 ng/mL mouse EGF and 2% FBS, or (6) EGF/PRL: 10 ng/mL EGF, 2 μg/mL ovinePRL and 2% FBS. The medium was refreshed every day.

### 3D culture and treatment of breast cancer (BC) cell lines

Similarly to MECs, 4000 BC cells (MCF7/WT, MCF7/SCR, MCF7/PRLR-KO and MDA-MB-231) in 100 μL growth medium were seeded on top of precoated and polymerized growth factor reduced Matrigel in 8-Chamber Cell Culture Slide. After 1.5 h, add 100 μL growth media containing 10% Matrigel to create a final concentration of 5% Matrigel in full growth medium. Cells were maintained in growth medium with 5% Matrigel for 2 d for mammosphere outgrowth. The morphology of the mammospheres was evaluated after 3 d of different treatments: (1) CTL: 2% FBS, (2) PRL: 250 ng/mL rhPRL and 2% FBS. The medium was refreshed every day.

### Immunofluorescence (IF) staining and confocal microscopy

For all 2D immunostaining experiments, cells were plated in 4 Chamber Cell Culture Slide (Celltreat, 229164) and were fixed in 4% PFA for 1 h at room temperature. Samples were permeabilized in 0.1% Triton X-100/PBS (PBST) for 15 min and blocked with 5% normal donkey serum in PBST for 1 h. Incubations with primary and secondary antibodies (listed in Supplementary Table S4) were done in the same buffer. Samples were mounted in ProLongTM Gold Antifade reagent with DAPI (Invitrogen, P36935). Samples were imaged on a Zeiss 780 LSM confocal microscope with an Axiovert 200 M microscope and a 20x/0.40 LD Plan-Neofluar. Zoom in when needed. Cells in 3D culture were fixed in 4% PFA for 1 h at room temperature, permeabilized in 0.3% PBST for 15 min and stained as described above. Samples were imaged on a Zeiss 780 LSM confocal microscope with an Axiovert 200 M microscope and a 63x/1.40 oil DIC Plan-Apochromat lens.

### RNA-Seq assay and analysis based on databases

Four RNA libraries from MCF7/SCR and four libraries from MCF7/PRLR-KO cells were sequenced at McGill Genome Centre with 100 bp stranded paired-end reads. Before mapping, raw reads were cleaned, trimmed and removed their adapter sequences. MultiQC package [52] was used for assessing read quality. Human genome (GRCh38) and annotation (version 44) files were downloaded from the Ensembl database. Reads with Phred score > Q30 were aligned using the splice-aware STAR aligner [53] with 2-pass alignments, according to the NCI Genomic Data Commons mRNA quantification analysis pipeline. STAR counts were compared with the htseq-count function from HTSeq [54]. Differential expression analysis was performed using DESeq2 [55], with low gene counts (< 10 reads) excluded. Differentiated Expressed Genes (DEG) with log2FC|>1.0| and adjusted *p*-value less than 0.05 were kept for downstream analysis. The Volcano plot was visualized using EnhancedVolcano package [https://bioconductor.org/packages/release/bioc/html/EnhancedVolcano.html]. Two-tailed Mann–Whitney *U*-test was performed to assess differences in gene expression profile between PRLR and CCN2 genes in MCF7/PRLR-KO RNA-seq libraries.

Gene Ontology terms and Human Wikipathways enrichment analyses were conducted [56], selecting the 20 top co-expressed genes with CCN2 (CTGF). Terms and pathways with an adjusted *p*-value, corrected for multiple hypotheses testing using the Benjamini-Hochberg method, less than 0.05 were considered significantly enriched. The Protein-Protein-Interaction network was generated using CCN2 symbol and physical interaction data from GeneMANIA [57]. The network interaction diagram was constructed using Cytoscape [58]. The expression profile and co-expression analysis of CCN2 and the polarity complex genes in BC were extracted from the Sweden Cancerome Analysis Network-Breast (SCAN-B) database (GSE202203), one of the most extensive population-based cohort of primary breast tumors to date [59]. Additionally, data from the microdissected breast epithelium (GSE141828) of healthy women [60] and the Molecular Taxonomy of BC International Consortium (METABRIC) database [9] were downloaded and processed using Python scripts. A pathway-centric co-expression analysis using Pearson correlation was performed, considering the relationship between PRLR and all genes associated with the Hippo signaling pathway (hsa04390) from the KEGG database using a threshold Pearson correlation with PRLR of > |0.3| [61]. Finally, co-expression data from TCGA and SRA human breast cancer were obtained from the Genefriends website [62] on July 10, 2024.

### Western blotting analysis

Cells were serum starved overnight and treated with or without 250 ng/mL ovine PRL for MECs or 250 ng/mL rhPRL for human cell lines in 10-cm dishes for indicated time as described in the text. Total protein lysates were obtained using RIPA lysis buffer (50 mM Tris pH 8, 150 mM sodium chloride, 1% NP-40, 0.5% sodium deoxycholate, 0.1% SDS, 1 mM Na3VO4 and Protease inhibitors cocktail). 30 µg proteins were loaded in the gel. Cell lysates were separated by electrophoresis in 8% sodium dodecyl sulfate-polyacrylamide gradient minigels (SDS-PAGE) and electrophoretically transferred to nitrocellulose membrane. Western blots were probed with the relevant primary antibodies and secondary antibodies listed in Table S4. Blots were visualized by enhanced chemiluminescence (ECL) under ChemiDoc (Bio-rad).

### RNA extraction and quantitative real-time PCR (qRT-PCR)

Total RNA was extracted from confluent BC cell lines MCF7 and MCF7/PRLR-KO with Trizol (Invitrogen, 15596018), according to the manufacturer’s instructions. Total RNA was reverse-transcribed with iScript reverse transcription supermix (Biorad, 1708840). Real-time monitoring of PCR amplification of the resulting cDNA was carried out for human CCN2 gene. Each sample was run in triplicates and normalized to GAPDH. Expression difference was assessed by 2^−ΔΔCt^ relative quantitative analysis. The primer sequences were as follows:

Human CCN2 forward AGACCCAACTATGATTAGAGCCA

Human CCN2 reverse TTGGAGATTTTGGGAGTACGGA

Human GAPDH forward GCCTCAAGATCATCAGCAGCAATGCCT

Human GAPDH reverse TGTGGTCATGAGTCCTTCCACGAT

### JAK2 and MST1 transient siRNA knockdown in primary MECs

The day before transfection, dissociate primary MECs were plated on 6-cm dishes or 4 Chamber Cell Culture Slide with 80–90% confluency. Silencer pre-designed siRNA against mouse JAK2 (4390771, S68539), MST1 (Am16708, 158044) and negative control (4390843) siRNAs were obtained from Thermo Fisher Scientific. Primary MECs were transfected with 50 nM of each siRNA using the lipofectamine 3000 (L3000008) protocol obtained from Thermo Fisher Scientific. After 48 h, cells were harvested for qRT-PCR and western blotting analysis or fixed in 4% PFA for IF staining.

#### Cellular fractionation/Nuclear extract

MDA-MB-231 cells were seeded as 1 × 10^6^ cells/dish into 10 cm dishes and grown in DMEM with 10% FBS until reaching to 60% confluency. The medium was changed to DMEM + 2% FBS with or without 250 ng/mL rhPRL for 0, 30, 60 or 180 min. The cells were lysed and collected in hypotonic buffer (10 mM HEPES-KOH pH 7.9, 1.5 mM MgCl_2_, 10 mM KCl, 20 mM NaF, 0.5 mM DTT, 1 mM Na3VO4, protease Inhibitor cocktail, phosphatase Inhibitor cocktail) on ice. After high-speed centrifugation, collect the supernatant as the cytosolic fraction. Add high salt buffer (20 mM HEPES-KOH pH 7.9, 1.5 mM MgCl_2_, 25% glycerol, 420 mM NaCl, 0.2 mM EDTA pH 8.0, 20 mM NaF, 1 mM Na3VO4, protease Inhibitor cocktail, phosphatase Inhibitor cocktail) to each pellet on ice. Vortex briefly at room temperature. Let sit on ice for 30 min vortexing every 5–10 min. After high-speed centrifugation, collect the supernatant as the nuclear fraction.

#### MTT assay

MDA-MB-231 cells were seeded triplicate as 4 × 10^3^ cells/100 μL/well into a 96-well plate and grown in DMEM with 10% FBS overnight. The medium was changed to DMEM + 2% FBS with 0.1 μM DMSO, 0.1 μM DMSO + 250 ng/mL rhPRL, 0.1 μM VPF or 0.1 μM VPF + 250 ng/mL rhPRL the day after and refresh the medium everyday. Then, the cells were incubated with 5 mg/mL 3-(4,5-dimethyl-2-thiazolyl)- 2,5-diphenyl-2H-tetrazolium bromide (MTT, Sigma, M2128) at 37 °C for 3 h after culture for 0 h, 24 h, 48 h,72 h. Nanodrop was used to measure the OD at 570 nm.

#### Wound healing assay

MDA-MB-231 cells were seeded as 1 × 10^5^ cells/well into 6-well plates and grown in DMEM with 10% FBS overnight. A straight scratch was obtained by 1000 uL pipette tips and the medium was changed to DMEM + 2% FBS with 0.1 μM DMSO, 0.1 μM DMSO + 250 ng/mL rhPRL, 0.1 μM VPF or 0.1 μM VPF + 250 ng/mL rhPRL the day after. The scratches were monitored by taking picture using Nikon NIS-Elements software at 0, 24 h, 48 h and 72 h. Refresh the medium each time after taking pictures. Measure the wound closure by ImageJ and calculate the ratio.

##### Statistics

The statistical analyses were performed using GraphPad Prism version 6 software. Significance was calculated using unpaired two-tailed Student’s *t*-test or ANOVA. Data are represented as mean ± SEM or ± SD with at least 3 independent experiments. The survival curves were evaluated using the Kaplan–Meier method with a log-rank test. Mann–Whitney *U*-test was performed to assess differences in gene expression profile between PRLR and CCN2 genes in MCF7/SCR and MCF7/PRLR-KO libraries. For all figures, statistical significance was represented as *p < 0.05, **p < 0.01 and ***p < 0.001.

## Results

### PRL regulates the apical-basal subcellular localization of the polarity protein complexes (Par, Crumb, and Scribble) enabling MECs acinar morphogenesis and functional differentiation

**Fig. 1:**
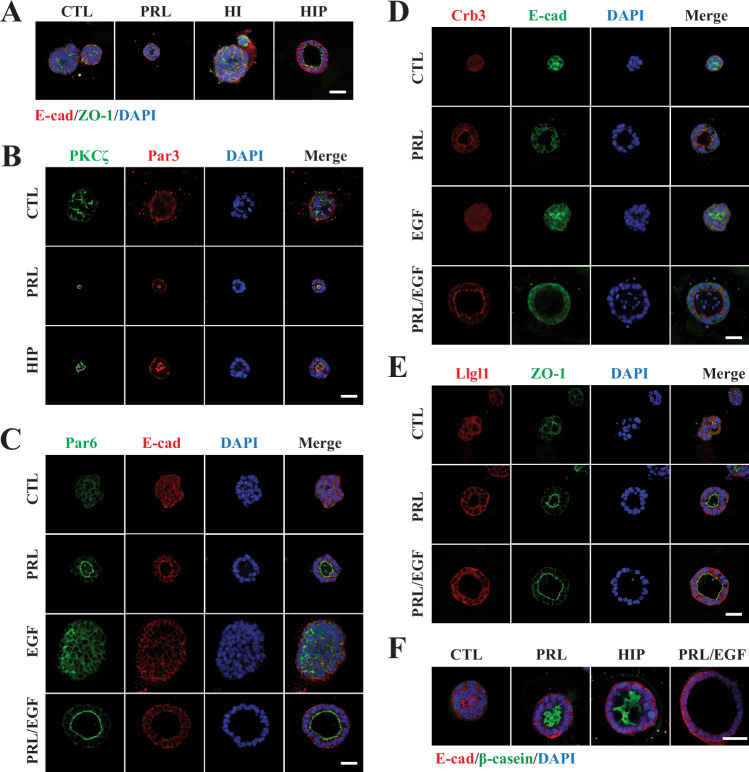
PRL-regulation of structural and functional differentiation of mammary acini and subcellular localization of polarity protein complexes. MECs ex-vivo grown as 3D cultures on Matrigel gel under different treatment conditions (CTL, HI, PRL, HIP, EGF or EGF/PRL) for three days and immuno-stained with various antibodies as indicated in figure legends. Nuclei were counter stained with DAPI (blue). Scale bar, 20 μm. **A** Acini formation under CTL, HI, PRL, HIP treatments were co-immuno-stained with antibodies to ZO-1(green) and E-cad (red). **B** Mammary acini under various treatments as indicated were co-immuno-stained with antibodies to PKCζ (green) and Par3 (red). **C** Mammary acini under various treatments as indicated were co-immuno-stained with antibodies to Par6 (green) and E-cad (red). **D** Mammary acini under various treatments as indicated were co-immuno-stained with antibodies to Crb3 (red) and E-cad (green). **E** Mammary acini under various treatments as indicated were co-immuno-stained with antibodies to Llgl1 (red) and ZO-1 (green). **F** Mammary acini under various treatments as indicated were co-immuno-stained with antibodies to β-casein (green) and E-cad (red).

Establishment of A/B polarity is a property of epithelial secretory organs and dysregulation of cell polarity is commonly observed in cancers such as breast cancer. Therefore, to better decipher the pro-differentiation role of PRL in the breast and its effect on breast tumorigenesis, we first examined the role of PRL in regulating polarity protein complexes in acinar morphogenesis. Previously, using ex-vivo 3-dimensional (3D) cellular culture models of primary mouse MECs of mid-gestation, we have shown that PRL induces acinar morphogenesis and the apical localization of zona occludens 1 (ZO-1) and the basal/lateral localization of E-cadherin (E-cad), consistent with the physiological role of PRL/PRLR signaling in mammary alveologenesis [43]. To expand on the role of PRL in regulating apical-basal polarity in MECs, we examined the role of PRL in regulating polarity protein complexes (Par, Crumb, and Scribble) using primary mouse MECs of the adult virgin state. As shown in Fig. 1A, cells grown in 3D culture were either left untreated (CTL) or treated with either PRL alone, hydrocortisone and insulin (HI) or with the lactogenic mix hydrocortisone, insulin and PRL (HIP) for a period of 72 h. Control or HI-treated MECs formed masses of cells with no distinct polarity and lacked characteristics of structured acini. On the other hand, PRL or HIP-treated cells formed acinar structures with a single lumen, surrounded by a single layer of A/B polarized epithelial cells, as seen by the apical localization of ZO-1 and the basal/lateral localization of E-cad. Quantification analysis revealed that PRL or HIP significantly increased acini formation by more than 70% compared to control (CTL) or HI-treated samples (Fig. S1A). Thus, our data identify PRL as a determinant cue of A/B polarity and a master regulator of mammary morphogenesis independent of the gestation milieu. As the establishment and maintenance of epithelial asymmetry is dependent on the polarity protein complexes, we next examined the subcellular localization of members of the Par, Crumb, and Scribble polarity protein complexes in response to PRL treatment in MECs. As can be seen in Fig. 1B, C, in contrast to CTL untreated cells, PRL or HIP exposure induced acinar morphogenesis with all 3 core Par complex proteins (PKCζ, Par3, and Par6) found to localize at the apical membrane domain surrounding the lumen. Moreover, as depicted in Fig. 1C, in Epidermal Growth Factor (EGF) treated MECs, acini morphogenesis was absent, and colonies lacked asymmetry in Par6 and E-cad localization. In contrast, combination treatment of EGF with PRL induced acini morphogenesis and caused Par6 to localize to the apical domain while E-cad localized to the basal/lateral domain, further emphasizing the morphogenic effects of PRL. Next, we investigated PRL regulation of the Crumb complex. Interestingly, as shown in Fig. 1D, in contrast with the diffuse staining of Crb3 in CTL cells, exposure to PRL alone or the combination of PRL and EGF activated morphogenesis programming and induced distinct apical localization of Crb3. In parallel, we also examined the subcellular localization of the Scribble complex (Llgl1 staining) known to define the basolateral domain in response to PRL treatment. As shown in Fig. 1E, in contrast to CTL samples, following treatments with PRL or combination of PRL and EGF, acini morphogenesis and polarization of Llgl1 to the basolateral membrane domain, whereas ZO-1 localization to the apical domain was observed. Together, these results indicate that PRL induces acinar morphogenesis and defines the apical and the basolateral domains through regulating the proper localization of the core polarity protein complexes in MECs. Furthermore, as the mammary acini ex-vivo mimics mammary alveolar morphogenesis that occurs in the mammary gland during lactation, the presence of the milk protein β-casein was assessed to evaluate the functional and terminal differentiation of the MECs. Notably, as shown in Fig. 1F, in contrast to cells in CTL conditions, the structured acini formed following treatments with PRL or HIP displayed intraluminal secretion of β-casein indicative of functional differentiation. As expected, acini formed under PRL and EGF co-treatment were deprived of β-casein expression in agreement with our previous data showing a negative crosstalk between PRL and EGF by which EGF blocks PRL-induced STAT5 activation of the β-casein gene promoter, whereas PRL blocks EGF-induced MEC growth [63] (Fig. S1). Overall, our results highlight the role of PRL in orchestrating the subcellular localization of the Par, Crumb, and Scribble polarity complexes, promoting A/B polarity and functional acini morphogenesis in MECs. This emphasizes the vital and broad differentiation effects of PRL in the breast.

### PRL/PRLR regulation of acinar morphogenesis and polarity gene network in breast cancer promoting patient survival outcomes

**Fig. 2:**
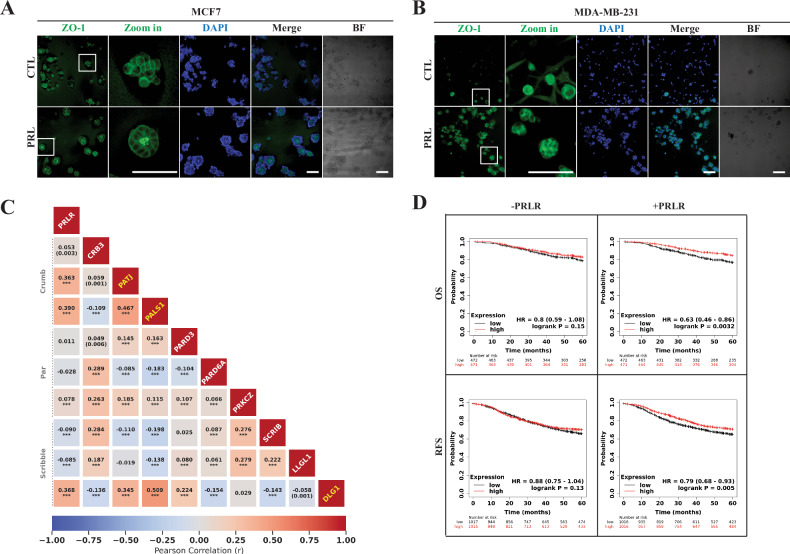
PRL/PRLR regulation of acinar morphogenesis and correlation with polarity proteins influencing patient outcome in breast cancer. Acinar morphogenesis in breast cancer cellular models MCF7 (**A**) and MDA-MB-231 (**B**) with or without PRL treatment. Individual cell lines were grown in a 3D culture on Matrigel and immuno-stained with antibodies to ZO-1(green). Nuclei were counterstained with DAPI (blue). Bright-field (BF) and zoom-in pictures are also shown. Scale bar, 20 μm. **C** Pearson pairwise correlation analysis comparing the gene expression profiles of polarity complexes (Crumb, Par, and Scribble) with the PRLR gene using the SCAN-B dataset. The Pearson correlation coefficient for each pairwise correlation is depicted, followed by its *q-*value, or a *p*-value *** <0.001 demonstrating the statistical significance of each gene pair. Pairwise correlations without their *q* values lack statistical significance. **D** Survival curves for PATJ, PALS1 and DLG1 genes, without or with the PRLR gene, using overall survival (OS) and relapse-free survival (RFS) as endpoints. The analysis included all cancer patient’s samples from the breast cancer Kaplan–Meier plotter database.

The central role of PRL in MEC differentiation suggests that it may also play a differentiation role in breast cancer, and this may contribute to its anti-tumorigenic effects. Having shown that PRL is a polarity cue in MECs, therefore, we investigated whether PRL may regulate acini morphogenesis in breast cancer cells representative of the different breast cancer subtypes when cultivated in our 3D-cell culture model. For this, we used various human breast cancer cell lines, including MCF7 (HR+), SKBR3 (HER2-enriched), MDA-MB-453 (TNBC- luminal/androgen receptor (LAR)), and MDA-MB-231 (TNBC-mesenchymal-claudin low). As observed in Fig. 2A, control MCF7 cells formed either small masses of cells and/or remained as single and flat cells. Interestingly, cell clusters with acini-like structures with apical localization of ZO-1 were uniformly observed in MCF7 cells treated with PRL. As well, these PRL-induced MCF-7 acini-like structures also showed basal/lateral localization of E-cad and diffused but discernible apical localization of Crb3 (Fig. S2A, B) On the other hand, both SKBR3 and MDA-MB-453 cells formed cell mass clusters in 3D cultures under control conditions and this cellular phenotype was maintained under PRL treatment with the exception that PRL treated SKBR3 cells formed smaller clusters (Fig. S2C, D). Interestingly whereas MDA-MB-231 cells, cultured without PRL, remained as single cells, however, in the presence of PRL clustering of cells with high expression of ZO-1was observed (Fig. 2B). Moreover, like the SKBR3 and MDA-MB-453, none of the MDA-MB-231 cell clusters exhibited acini-like structures and ZO-1 did not show apical localization. Together, these results show that among the different breast cancer subtypes, PRL-induced acinar-like structures predominantly in the most differentiated HR+ breast cancer cells and promoted the elaboration of epithelial-like features (cell–cell clustering) in cells representing the mesenchymal claudin-low TNBC subtype implicating a morphogenic and differentiation role for PRL in breast cancer.

To expand our understanding of the differentiation role of PRL in breast cancer in relation to the A/B polarity players, we next employed bioinformatics analysis. We examined the correlation in gene expression of the core polarity proteins together as well as in relation to PRLR gene expression in the Sweden Cancerome Analysis Network-Breast (SCAN-B) database (GSE202203) containing a large RNA-Seq dataset of 3207 breast cancer patients, making it one of the most extensive population-based cohorts of primary breast tumors to date. Figure 2C shows a heatmap Pearson pairwise correlation analysis, which allowed us to identify a co-expression gene network among the core polarity proteins and the PRLR gene. Our analysis revealed that, within the Crumb complex, PATJ and PALS1 showed the highest positive correlation (*r* = 0.467). The DLG1 gene was the most significant positive correlation with PALS1 (*r* = 0.509) and PATJ (*r* = 0.345). Interestingly, the expression of these three genes (PATJ, PALS1, and DLG1) also showed the most positive correlation with PRLR gene expression (*r* = 0.363, *r* = 0.390, and *r* = 0.368, respectively). On the other hand, our heatmap correlation analysis revealed that within the Par and the Scribble complexes, there were generally weak correlations in gene expressions. In all these correlation analyses, a cutoff of 0.3 (a moderate correlation) and *q*-value was used. Considering we have identified a co-expression gene network of polarity proteins, we next investigated their clinical impact in breast cancer patients using the Kaplan–Meier plotter database [64]. Interestingly, while no significant impact on patient outcomes was observed for 5-years relapse-free survival (RFS) (*n* = 2032, *p* = 0.13), overall survival (OS) (*n* = 943, *p* = 0.15), or distant metastasis-free survival (DMFS) (*n* = 958, *p* = 0.25) for these highest correlated polarity genes (PATJ, PALS1, and DLG1), the inclusion of the PRLR gene significantly improved survival outcomes for both RFS (*n* = 2032, HR = 0.79, *p* = 0.005) and OS (*n* = 943, HR = 0.65, *p* = 0.0032) (Figs. 2D and S3). These findings highlight that co-expression of PRLR along with polarity proteins positively promotes patient survival outcomes within the wider breast cancer population emphasizing the essential and clinical relevance of contribution of PRL/PRLR pathway in driving a positive prognostic value of A/B polarity proteins on breast cancer patient outcome.

### Loss of PRLR expression in HR+ breast cancer cells revealed CCN2 as the top upregulated gene and their antagonistic relation was validated in mammary and large breast cancer datasets

**Fig. 3:**
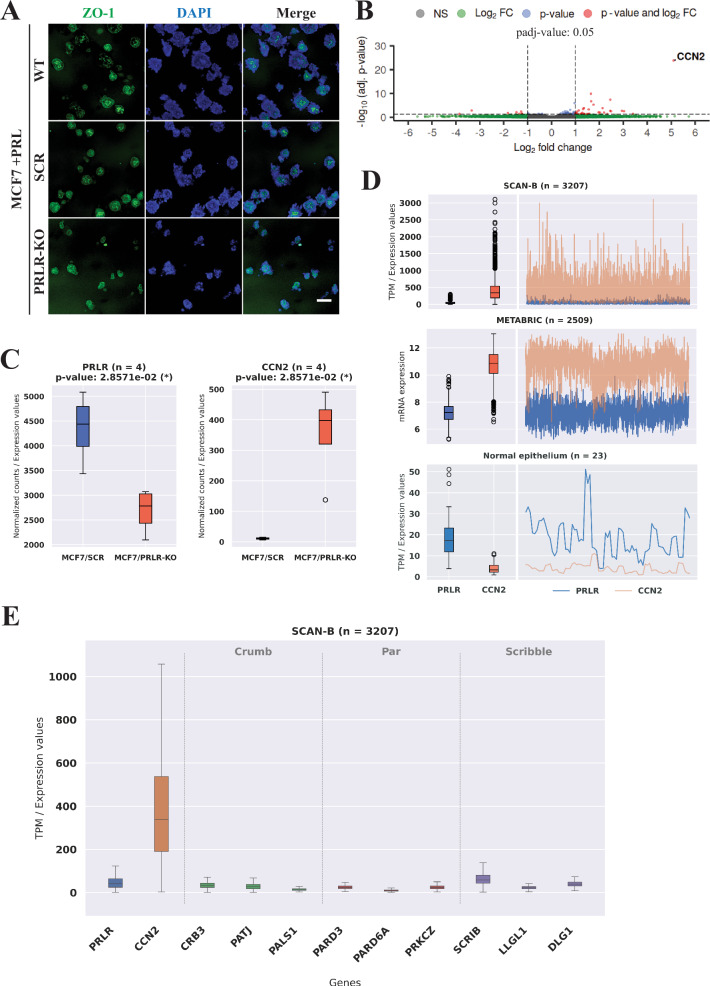
CRISPR/Cas9 knockout of the PRLR in HR+ breast cancer cells disrupt acini morphogenesis and defines antagonistic relationship with CCN2 gene. **A** Acinar morphogenesis in MCF7 wild type (WT), MCF7/scramble (SCR) and MCF7/PRLR-KO cells with PRL treatment. Individual cell lines were grown in a 3D-culture on Matrigel and immuno-stained with antibodies to ZO-1(green) and nuclei were counterstained with DAPI (blue). Scale bar, 100 μm. **B** Volcano plot illustrates the results of the DEseq2 analysis for Differentially Expressed Genes (DEGs) from MCF7 SCR and PRLR-KO libraries, with a log2FC cut-off of > |1| and an adjusted *p*-value cut-off of 0.05. Genes are color-coded: gray for non-significant genes, green for genes outside the log_2_FC range of -1 to 1 but statistically non-significant (*p* ≥ 0.05), blue for statistically significant genes (p < 0.05) but outside the log2FC range, and red for genes that are both statistically and log_2_FC significant. Abbreviations: NS (Non-Significant), FC (Fold Change). **C** PRLR and CCN2 gene expression levels in MCF7/SCR and MCF7/PRLR-KO RNA-seq libraries obtained from normalized counts using the DESEq2 package. *p*-values were obtained using the Mann–Whitney *U*-test. **D** The expression of PRLR and CCN2 genes in Transcription per Million (TPM) were analyzed in two distinct breast cancer datasets and microdissected normal breast epithelium with sample numbers (n) as indicated. Boxplots consolidate visually both gene expressions within each dataset, while line graphs illustrate their expressions across the samples. **E** Boxplot comparative view of gene expression of PRLR and CCN2 measured in TPM excluding extreme gene expressions, along with genes associated with Crumb (CRB3, PATJ, PALS1), Par (PARD3, PARD6A, PRKCZ) and Scribble (SCRIB, LLGL1, DLG1) polarity protein complexes in the SCAN-B dataset.

Our previous results emphasized PRL/PRLR pathway as a promoter of differentiation in breast cancer cells driving favorable patient outcomes. To further dissect the role and contribution of the PRL/PRLR pathway in regulating differentiation in breast cancer, we made use of the CRISPR/Cas9 knockout of the PRLR in MCF7 cells (MCF7/PRLR-KO) previously generated [51]. Proper PRLR gene silencing was ensured and validated by western blotting (Fig. S4A) and Surveyor assay (Fig. S4B). As shown in Fig. 3A, PRL treatment of MCF7 wild type (WT), scramble (SCR) and PRLR-KO cells cultured as 3D-cell cultures formed acini-like structures and promoted the apical localization of ZO-1 in both WT and SCR cells but not in PRLR-KO cells. The MCF7/PRLR-KO cells grew as small clusters and did not show acinar phenotype and presented reduced ZO-1 expression, confirming that PRLR expression is critical in driving acinar morphogenesis in HR+ breast cancer cells. Notably, these results also emphasize and substantiate our previous findings demonstrating the substantial dedifferentiation consequences caused by loss of PRLR gene expression in these HR+ breast cancer cells encompassing increased cellular luminal-basal-mesenchymal plasticity, stemness and increased tumorigenicity [51]. To take advantage of this model and identify target genes and pathways that may play a role in PRL-induced differentiation, we generated bulk RNA-seq libraries of the MCF7/SCR and the MCF7/PRLR-KO cells. The analysis of the RNA-seq data showed an average of 96% uniquely mapped reads per sample (Fig. S4C, D) and identified the gene CCN2 as the highest differentially expressed (log_2_FC > 5, adjusted *p*-value = 1.50E-024) between SCR and PRLR-KO cells (Fig. 3B). These results revealed that loss of PRLR in MCF7 cells is associated with an increase in mRNA expression of CCN2 gene (Fig. 3C). To build upon these results and examine its validity in human breast cancer tumor samples, we investigated PRLR and CCN2 gene expression profiles in the breast cancer SCAN-B (*n* = 3207) and METABRIC (*n* = 1992) cohorts (Fig. 3D, upper and middle panels). Significantly, our results revealed distinctive gene expression patterns of PRLR and CCN2, with PRLR gene exhibiting a low expression level among all breast cancer cases compared to that of CCN2 gene in both datasets, corroborating our RNA-seq analysis and emphasizing the inverse relation in gene expression between PRLR and CCN2 in breast cancer. To further validate this conclusion, we next sought to evaluate the expression profile of PRLR and CCN2 in normal human breast tissue. For this, we used RNA-seq data of microdissected mammary epithelium tissue (*n* = 23). This microdissected RNA-seq dataset provides us with a more precise characterization of tissue-specific PRLR gene expression patterns compared to other bulk non-microdissected RNA datasets (Fig. S5). Interestingly, we found that the expression dynamics for PRLR and CCN2 genes shifted in comparison to that seen in breast cancer tumor samples where PRLR in these microdissected normal mammary epithelial samples showed high expression levels while CCN2 exhibited low expression (Fig. 3D, lower panel). As MCF7/PRLR-KO cells showed loss of acinar morphogenesis and cell polarity, we next compared the expression profile of PRLR and CCN2 genes with all other genes belonging to the core polarity protein complexes in the SCAN-B dataset (Figs. 3E and S6). The CCN2 TPM exhibited an interquartile range (which 50% of the data falls) from around 200 (Q1) up to 500 (Q3), whereas all other analyzed genes showed a median value below 50 TPM. The data illustrates that the mRNA expression levels of members of the Crumb, Par, and Scribble polarity protein complexes, like the pattern of PRLR gene expression, were all low compared to the expression of the CCN2 gene (Fig. 3E). In summary, these results spotlight the inverse relationship between PRLR and CCN2 gene expression in breast cancer tumors and normal mammary tissue as well as emphasize that the CCN2 gene is a downstream target of PRLR and potentially may play a role in disrupting PRL-induced differentiation and cellular A/B polarity.

### Bioinformatics and functional validation of a link between PRL signaling and Hippo-YAP pathway in breast cancer

**Fig. 4:**
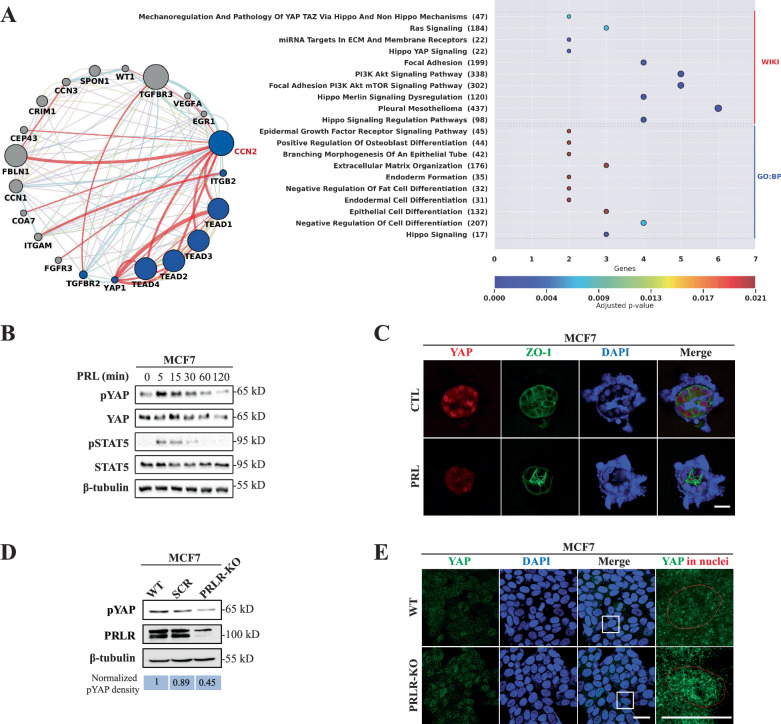
PRLR and Hippo pathway gene expression and interaction network in breast cancer impacts patient outcome. **A** Left panel, a gene–gene interaction network for the CCN2 gene was constructed using GeneMania and visualized through Cytoscape application. In the network, blue nodes represent genes associated with the Hippo pathway, while the red edge thickness signifies the strength of their physical interactions. The other edge colors show different types of interactions, including co-expression, co-localization, genetic interactions, pathway interactions, and shared protein domains. Right panel, the graphical representation illustrates Gene Ontology and pathway enrichment analyses performed using Enrichr on the top 20 enriched genes associated with the CCN2 gene. Biological processes (GO:BP) and WIKI are depicted, where the x-axis represents the number of genes with their adjusted *p*-values enriched in each pathway or Gene Ontology term. The y-axis represents GO:BP terms and WIKI with the pathway names followed by the total number of genes in each respective term or pathway. **B** Immunoblot analysis of YAP phosphorylation (pYAP) in response to PRL treatment in MCF7 cells. **C** MCF7 cells were grown as 3D culture and co-immuno-stained with antibodies to YAP (red) and ZO-1 (green). Nuclei were counterstained with DAPI (blue). Scale bar, 20 μm. **D** Western blot analysis of PRLR and pYAP levels in MCF7 WT, SCR and PRLR-KO cells. Relative value of pYAP was normalized by β-tubulin. **E** MCF7 WT and MCF7/PRLR-KO cells were cultured as monolayer, and immuno-stained with antibody to YAP (green) and nuclei were counterstained with DAPI (blue). Scale bar, 20 μm.

To elucidate the role CCN2 in regulating/altering PRL/PRLR effects in mammary and breast cancer cells, we first generated CCN2 physical interaction gene network (Materials and Methods) (Fig. 4A, left panel). Next, we conducted pathway enrichment analyses (WIKI) and Gene Ontology Biological Process (GO:BP) associated with the top twenty CCN2 co-expressed genes identified by Enrich and the top ten significant pathways and GO terms were identified (Fig. 4A, right panel). As expected, the results demonstrated that CCN2 gene physically interacts with several genes from the Hippo signaling pathway, such as TEAD1-4 and YAP1, and two genes from the Transforming Growth Factor-beta (TGF-β) pathway. In addition, according to the pathway enrichment results, the most enriched pathways are also associated with Hippo signaling. Moreover, GO terms identified CCN2 to be involved in diverse biological processes, notably epithelial cell differentiation. All enriched GO terms and pathways presented adjusted *p*-values below 0.05. These analyses emphasize CCN2 gene as part of the Hippo pathway and implicate Hippo pathway in regulating PRL differentiation effects in breast cancer.

To validate the implication of these bioinformatics results, we evaluated PRL’s role in activating the Hippo pathway in the HR+ breast cancer cells MCF7. Interestingly, when treated with PRL, MCF7 cells exhibited a rapid increase (within minutes) in the phosphorylation of YAP at Ser-127 suggesting Hippo as a downstream pathway of PRL signaling in breast cancer cells (Fig. 4B). To further examine the ability of PRL to regulate YAP activity, we examined YAP localization following PRL-induced acini morphogenesis in MCF7 3D-cell culture models. Importantly, we found strong nuclear localization of YAP in MCF7 3D-cell clusters in the absence of PRL, even in rare, organized acini-like structures. Whereas, upon PRL treatment, YAP showed low nuclear accumulation and loss in overall protein levels (Fig. 4C). To further elaborate on the role of PRL signaling in regulating YAP, we examined YAP phosphorylation and nuclear accumulation in control MCF7 cells (parental and SCR) in comparison to MCF7/PRLR-KO cells. Indeed, MCF7/PRLR-KO cells showed loss of YAP phosphorylation (Fig. 4D) in comparison to control cells and increased overall YAP immunofluorescence intensity throughout the cells and notably caused nuclear accumulation of YAP in comparison to control cells (Fig. 4E). Altogether, based on our bioinformatics analysis and functional validation experiments these results established a link between PRL/PRLR and Hippo pathway and as an important negative regulator of YAP-CCN2 pathway in breast cancer.

### PRL/JAK2 activation induces Hippo pathway and nuclear exclusion of YAP in MECs critical for acinar morphogenesis and establishment of cell-cell junctions

**Fig. 5:**
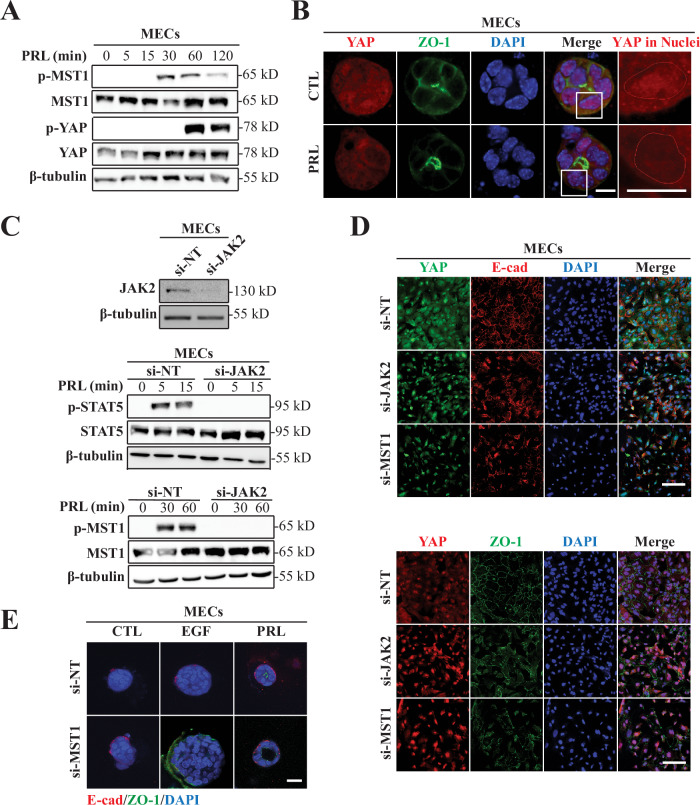
The role of PRL/PRLR/JAK2 axis in acinar morphogenesis and Hippo pathway activation in mammary epithelial cells. **A** Immunoblot analysis of total cell lysates of primary mouse MECs using antibodies to phospho-MST1 and phosphor-YAP following PRL stimulation for the indicated times. **B** MECs ex-vivo grown as 3D cultures on Matrigel were either untreated or treated with PRL for 3 days and co-immuno-stained with antibodies to YAP (red) and ZO-1 (green). Nuclei were counter stained with DAPI (blue). Red circle indicates the edge of a nucleus based on DAPI staining and red staining represents nuclear YAP. Scale bar, 20 μm. **C** Primary MECs were transfected with either si-NT (siRNA non-targeting) or si-JAK2 (siRNA targeting JAK2) and immunoblot analysis of JAK2 were carried out (upper panel) confirming knockdown of JAK2. Total cell lysates of si-NT and si-JAK2 of primary MECs following PRL stimulation for the indicated times were immunodetected using antibodies to phospho-STAT5 and phospho-MST1 (lower 2 panels). **D** Primary MECs were transfected with either si-NT, si-JAK2 or si-MST1 (siRNA targeting MST1). Cells were then co-immuno-stained with antibodies to YAP (green) and E-cad (red) (upper panel) or co-immuno-stained with antibodies to YAP (red) and ZO-1 (green) (lower panel). Nuclei were counter stained with DAPI (blue). **E** Primary MECs transfected with si-NT or si-MST1 were grown under 3D culture conditions and were either left untreated (CTL) or treated with EGF or PRL for 3 days and co-immuno-stained stained with antibodies to ZO-1 (green) and E-cad (red). Nuclei were counter stained with DAPI (blue). Scale bar, 20 μm.

Having shown that PRL/PRLR pathway regulates Hippo/YAP pathway in breast cancer cells, we next wished to determine whether PRL signaling also exerts a regulatory role on the Hippo pathway in MECs and whether this regulation is linked to PRL-mediated mammary acini morphogenesis. Indeed, treatment of MECs with PRL resulted in the phosphorylation of MST1 within 30 min, with subsequent YAP phosphorylation, providing evidence that PRL activates the canonical Hippo signaling pathway in MECs (Fig. 5A). The ability of PRL to activate Hippo pathway was also seen in PRL-induced acini of MEC, where we observed a clear nuclear exclusion of YAP upon PRL treatment (Fig. 5B). To delineate PRL’s signaling mechanism regulating this phenomenon, JAK2 kinase, a principal kinase downstream of the PRLR, was knocked down using siRNA (si-JAK2) in primary MECs (Fig. 5C, upper panel). Compared to control group transfected with non-targeting siRNA (si-NT), si-JAK2 MECs exhibited unresponsiveness to PRL stimulation, as evidenced by their inability to phosphorylate STAT5 (Fig. 5C, middle panel). Interestingly, PRL also failed to induce activation of MST1 in si-JAK2 MECs, underlying the indispensable role of the PRL/PRLR/JAK2 signaling cascade in Hippo pathway activation in MECs (Fig. 5C, lower panel). Next, we examined whether loss of JAK2 expression in MECs would affect YAP nuclear localization. Remarkably, si-NT transfected cells showed diffuse YAP staining throughout (cytoplasm and nuclear) with well established adherence (membrane localization of E-cad) (Fig. 5D, upper panel and Fig. S7A) and tight junctions (membrane localization of ZO-1) (Fig. 5D, lower panel and Fig. S7A), forming a well-defined basket-like structure. In contrast, si-JAK2 transfected MECs, like si-MST1 transfected MECs (Fig. S7B) showed strong nuclear YAP localization which was associated with the disruption of the intercellular adhesion and junctional structures (Fig. 5D, upper and lower panels and Fig. S7A). These results together underscore the crucial roles of PRL/JAK2 signaling in regulating Hippo/YAP pathway promoting proper epithelial cell-cell adhesion and junctional complexes. We next investigated the ability of si-JAK2 MECs (Fig. S7C) and si-MST1 MECs (Fig. 5E) to form acini structures in response to PRL treatment. As expected, 3D cultures of si-NT MECs grew as colonies of cell masses (unorganized colonies) in CTL (2%FBS) or in the presence of the growth factor EGF. Whereas they showed polarized, acini structures upon treatment with PRL or HIP. Conversely, si-JAK2 MECs as well as si-MST1 MECs were unable to form acinar structures, lacked distinct polarization showing irregular E-cad and ZO-1 staining, under all conditions tested (Figs. S7C and 5E). Notably, JAK2 knockdown as well as MST1 knockdown sensitized the MECs to EGF, resulting in significantly larger clusters compared to the si-NT MECs after EGF treatment (Figs. S7C and 5E). Altogether, these results establish a novel link between PRL/JAK2 signaling and Hippo pathway and define the critical role of PRL/PRLR/JAK2 cascade in activating Hippo/YAP pathway in MECs critical for mediating PRL differentiation effects in acini morphogenesis and cell-cell junctions.

### Blocking YAP-CCN2 pathway with verteporfin re-establishes a differentiation phenotype mimicking expression of PRLR in HR+ breast cancer cells

**Fig. 6:**
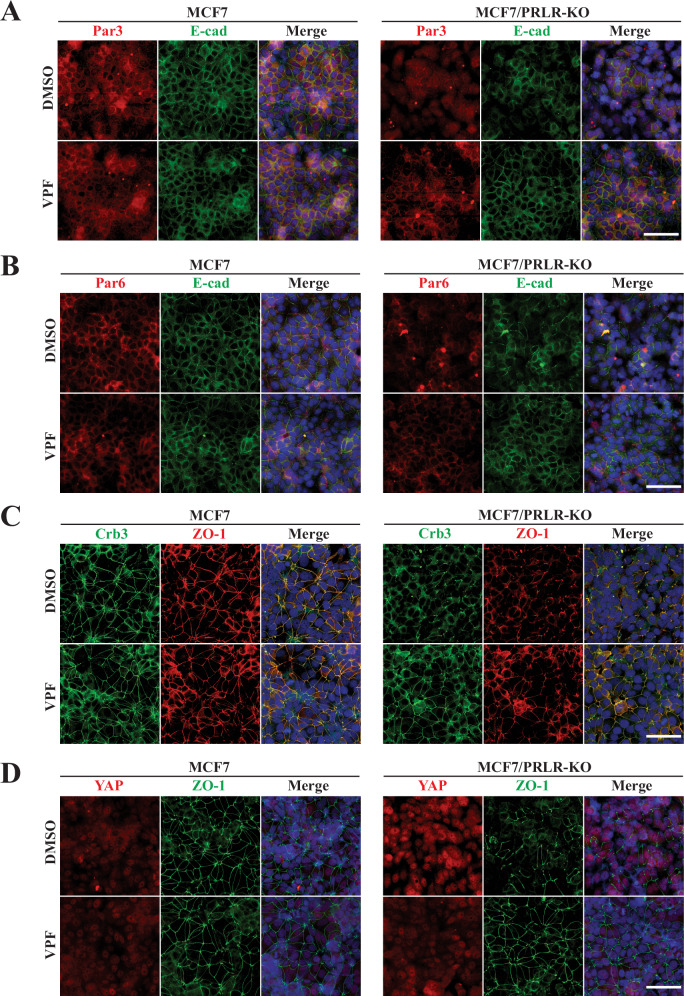
VPF rescues the proper subcellular localization of polarity protein complexes and cell-cell adherence and tight junctions in MCF7/PRLR-KO cells. **A–D** MCF7 WT and MCF7/PRLR-KO cells were cultured as monolayer and untreated or treated with 0.1 μM VPF for 48 h. Cells were then co-immuno-stained with various antibodies as indicated in figure legend. Nuclei were counter stained with DAPI (blue). Scale bar, 100 μm.

**Fig. 7:**
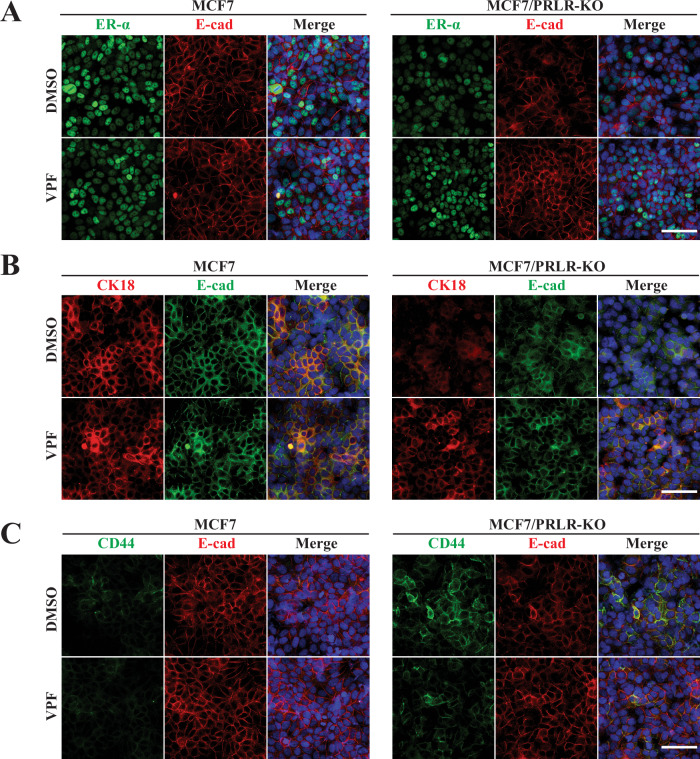
VPF rescues luminal phenotype and suppresses stem-cell marker expression in MCF7/PRLR-KO cells. **A–C** MCF7 WT and MCF7/PRLR-KO cells were cultured as monolayer and untreated or treated with 0.1 μM VPF for 48 h. Cells were then co-immuno-stained with various antibodies as indicated in figure legend. Nuclei were counter stained with DAPI (blue). Scale bar, 100 μm.

The above results emphasized that PRL/PRLR/JAK2-induced differentiation programming in mammary and breast cancer cells requires Hippo activation and YAP nuclear exclusion. Thus, we hypothesized that inhibitors of the YAP-CCN2 cascade would restore differentiation. To test this hypothesis, we made use the FDA approved pharmacological inhibitor of YAP verteporfin (VPF), known for its effect to block YAP-TEAD interaction and block YAP transcriptional program and downstream associated pathways, including CCN2 expression. Indeed, in agreement with our RNA-Seq data, CCN2 expression levels in MCF7/PRLR-KO cells were significantly higher (6-fold) compared to parental MCF7 cells and that treatment with VPF resulted in a significant reduction in CCN2 expression levels in both parental and KO cells (Fig. S9). Under these conditions, we first examined the ability of VPF to restore intercellular adherence and tight junctions by examining the membrane localization of E-cad, Par3, Par6, Crb3, and ZO-1. As shown in Fig. 6A–C, treatment of MCF7/PRLR-KO cells with VPF reinstated proper cellular adhesion, polarity and tight junction proteins to the cell membrane, like what was observed in parental cells treated with vehicle alone. These effects were also correlated with loss of YAP nuclear levels in MCF7/PRLR-KO cells following treatment with VPF (Fig. 6D). An important effect of loss of PRLR expression in HR + MCF7 breast cancer cells were the promotion of luminal-to-basal conversion as measured by the loss of expression of the luminal markers CK18 and ER and the gain in stem cell marker CD44 expression. Therefore, here we investigated whether blocking YAP-CCN2 would revert this tumorigenic phenotype. Remarkably, treatment with VPF restored ER and CK18 expression, whereas it suppressed CD44 expression in MCF7/PRLR-KO cells (Fig. 7A–C). Together, our results revealed that loss of PRLR expression in HR + MCF7 cells resulted in loss of cell-cell junctional complexes and a shift from luminal to basal-stem like cellular plasticity and that blocking YAP-CCN2 signaling with the chemical inhibitor VPF reestablished proper intercellular junctional adherence and luminal differentiation phenotype in breast cancer cells deficient in PRLR expression. These results further demonstrate a functional antagonistic relationship between PRL/PRLR/Hippo pathway and the oncogenic YAP-CCN2 pathway, limiting cellular plasticity and stemness and preserving cellular structural and architectural integrity.

### Translational impact of PRL/PRLR antagonistic effects on YAP-CCN2 pathway in breast cancer

**Fig. 8:**
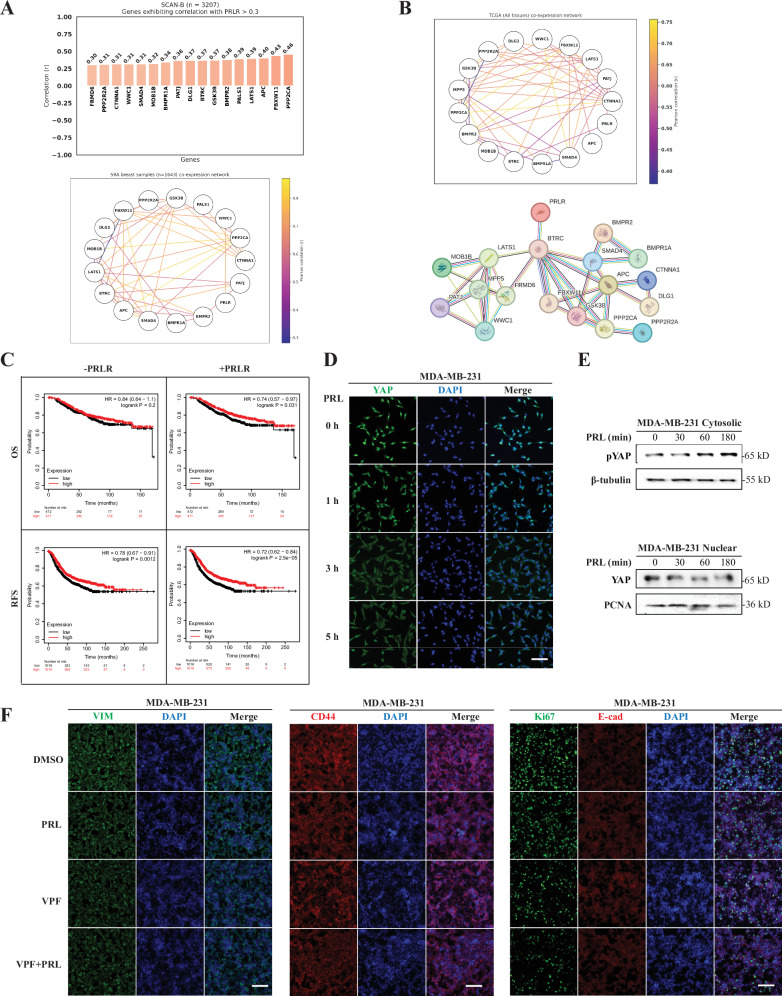
Clinical and preclinical significance of PRL/PRLR/Hippo/polarity pathway in breast cancer and other cancer types. **A** Upper panel bar plot displays the Pearson correlation coefficients of a pairwise correlation analysis between the PRLR gene and genes belonging to the KEGG Hippo pathway (hsa04390). Only correlations with coefficients greater than or equal to 0.3 were included in the graph. Lower panel presents a gene co-expression map derived from 1643 RNA-seq samples of human breast cancers from Sequence Read Archive (SRA) RNA-seq samples. The analysis was conducted using the Genefriends tool and focused on 17 genes selected from the Hippo pathway (hsa04390) that exhibited a positive correlation (r ≥ 0.3) with the PRLR gene. The colors of the edges correspond to the Pearson correlation coefficients between the genes. **B** This network represents gene co-expression analysis derived from the Cancer Genome Atlas Program (TCGA) from 10544 RNA-seq samples. The analysis focused on 17 genes selected from the Hippo pathway (hsa04390) that exhibited a positive correlation (*r* ≥ 0.3) with the PRLR gene. The analysis was conducted using the Genefriends tool encompassing TCGA data from 33 cancer types. The colors of the edges in the network correspond to the Pearson correlation coefficient between genes. Lower panel displays a STRING-DB protein-protein interaction (PPI) network generated for the 17 genes (above) that are positively correlated with PRLR. The PPI enrichment *p*-value for this network is < 1.0e-16. Proteins are represented by nodes (colored circles), and their interactions are denoted by colored lines between nodes (edges). Red edges indicate interactions supported by genetic evidence, green edges represent neighborhood relationships between proteins, blue edges indicate co-occurrence across species, purple edges represent experimental evidence supporting the interaction, yellow edges signify interactions inferred from text mining of literature abstracts, light blue edges denote interactions supported by curated databases, and black edges represent co-expression of proteins in the same or other species. **C** Survival curves for all 17 genes listed in (**A**) without and with the PRLR gene, using overall survival (OS) and relapse-free survival (RFS) as endpoints. The analysis included all cancer patient’s samples from the breast cancer Kaplan–Meier plotter database with follow up threshold of 180 months. **D** MDA-MB-231 cells were cultured as monolayer with 250 ng/mL PRL for 0, 1, 3 or 5 h (h) and immuno-stained with antibody to YAP (green). Nuclei were counterstained with DAPI (blue). Scale bar, 20 μm. **E** Cytosolic/nuclear cellular extracts from MDA-MB-231 were prepared following PRL treatment as indicated. Western blotting was performed antibodies to pYAP, YAP, β-tubulin, and PCNA as indicated. **F** MDA-MB-231 cells were cultured as monolayer and treated with vehicle (0.01% DMSO), 0.1 μM VPF, 250 ng/mL PRL or both for 48 h and immuno-stained with antibody to VIM, CD44, Ki67 or E-cad (as indicated). Nuclei were counterstained with DAPI (blue). Scale bar, 20 μm.

Having demonstrated a molecular antagonism between PRL/PRLR pathway and YAP-CCN2 pathway in mammary and breast cancer cells, we next sought to determine the clinical impact of this antagonism regarding breast cancer prognosis and potential therapeutic value. We first investigated the link between PRLR gene expression and expression of members of the Hippo pathway as defined by the Kyoto Encyclopedia of Genes and Genomes (KEGG) Hippo signaling pathway. This KEGG pathway includes adherence junction (hsa04520), tight junction (hsa04530), and genes from the polarity protein complexes as well as other pathways such as Wnt signaling pathway (hsa04310), and TGF-β signaling pathway (hsa04350) as well as pathways related to ubiquitin-mediated proteolysis (hsa04120) (Fig. S8A). We retrieved 158 genes from the KEGG database and performed a pathway-centric co-expression analysis with PRLR gene using Pearson correlation in the SCAN-B breast cancer dataset. Remarkably, PRLR expression positively correlated with genes in almost all pathways within the KEGG Hippo signaling pathway (Fig. S8B and Table S1). This analysis found 17 genes from the Hippo pathway to be highly co-expressed with the PRLR gene (r ≥ 0.3) such as LATS1, MOB1B (core Hippo pathway components), WWC1 and FRMD6 (known to link polarity proteins to Hippo pathway) and the polarity proteins (PALS1, DLG1 and PATJ). To explore the relationship and interactions between these genes based on their expression profiles in other datasets, a gene co-expression network was generated using the Genefriends tool. The Sequence Read Archive (SRA) from the human breast cancer co-expression map, which comprises 1643 RNA-seq samples (Fig. 8A lower panel), was used as reference. The map revealed a highly interconnected co-expression network, presenting a moderate to high Pearson correlation (r ≥ 0.5), suggesting a potential biological relationship between these genes. Additionally, the same analysis was performed using the TCGA dataset. Figure 8B upper panel presents a co-expression map derived from 10,544 RNA-seq samples covering 33 cancer types, including breast invasive carcinoma (*n* = 1134), kidney renal clear cell carcinoma (*n* = 544), lung adenocarcinoma (*n* = 542), colon adenocarcinoma (n = 505), head and neck squamous cell carcinoma (*n* = 504), among others (Table S2). The network showed Pearson correlations (*r* ≥ 0.4), highlighting the interplay between these genes. Gene co-expression network offers valuable insights into identifying functionally related genes. However, it reflects regulation at the mRNA level and not at the protein level. Therefore, a protein-protein interaction network was constructed using STRING-DB, a web resource to predict protein-protein interactions (PPIs) containing various published and biologically validated sources, to examine whether PRLR forms an interaction network with the above mentioned 17 positively correlated genes. Indeed, our analysis revealed that PRLR forms a PPI network with the KEGG Hippo genes through the gene BTRC. The interaction between the PRLR and the BTRC genes in the PPI network (Fig. 8B, lower panel) has a combined score of 0.689. This score is supported by experimental/biochemical data, associations in curated databases, and co-mentions in PubMed abstracts. Having shown that PRLR gene forms a co-expression network with components of the Hippo/polarity pathway, we next investigated whether this co-expression network affect patient outcome. As shown in the upper panel of Fig. 8C, patient outcomes were significantly improved when incorporating the PRLR gene along with the selected 17 Hippo/polarity genes. This improvement was observed in both OS, with an increase in patient lifespan of over 40 months, and RFS analyses resulted in an extended relapse-free time of over 16 months (Table S3). Together, these results highlight a crucial interconnection between PRLR expression and the anti-tumorigenic Hippo/polarity pathway, demonstrating its significant and vital positive clinical impact on patient outcomes in breast cancer.

As the antagonistic relationship between PRLR expression and CCN2 is observed in large breast cancer datasets and PRLR/Hippo gene signature marks breast cancer patients with favorable prognosis, therefore, we hypothesized that PRL along with agents that block YAP-CCN2 cascade could have value as single and/or combination therapies in breast cancer through restoration of differentiation and thereby limiting tumor cell growth. To examine this hypothesis, we made use of the MDA-MB-231 cells a well established TNBC preclinical cell model. First, we examined whether PRL treatment of MDA-MB-231 cells regulated YAP function. Indeed, PRL stimulation of MDA-MB-231 cells caused YAP phosphorylation as well as STAT5 phosphorylation, as control (Fig. S10A). Interestingly, we also found that PRL stimulation of MDA-MB-231 cells altered the intracellular localization of YAP. Indeed, in CTL untreated condition, we observed a strong nuclear immunostaining of YAP. This pattern was also seen at 1 hr post PRL stimulation. Interestingly at 3 h and 5 h post PRL treatment we observed nuclear clearing and mostly cytosolic staining of YAP (Fig. 8D). PRL-regulation of YAP nuclear exclusion was also confirmed using cell fractionation and western blotting (Fig. 8E). Treatment of MDA-MB-231 cells with PRL resulted in cytosolic accumulation of phospho-YAP with concurrent decrease in nuclear YAP. These results emphasize PRL as an extracellular ligand function to activate Hippo pathway, causing YAP phosphorylation and its nuclear exclusion suppressing YAP function in breast cancer cells. Finally, we investigated whether PRL and the pharmacological YAP inhibitor VPF can promote differentiation and suppress cell growth in MDA-MB-231 cells. Notably, as seen in Fig. 8F, each treatment individually suppressed mesenchymal (vimentin, abbreviated as VIM) and stemness markers (CD44) levels and when combined produced more pronounced effects. Importantly, each treatment also caused suppression of the proliferative marker Ki67 and indeed when combined caused superior suppressive effects. Interestingly, under these treatment conditions we also observed rescue in E-cad expression (Fig. 8F). Next, performed cell viability (MTT) and cell migration (Wound healing) assays following treatments with either PRL, VPF or PRL + VPF. Importantly, our results showed that both assays while each treatment alone suppressed cell viability and cell migration the combination treatment of PRL + VPF showed the most significant suppressive effects (Figure S10B, C).

These results highlight PRL, VPF and their combination treatment in promoting differentiation and anti-proliferative effects in the aggressive breast cancer cell model MDA-MB-231. Altogether, this data offers compelling evidence of the clinical prognostic value of the antagonistic relation of the pro-differentiation PRL/PRLR/Hippo/polarity protein complexes and further provide preclinical evidence of a therapeutic value of PRL, YAP inhibitor and their combination as a differentiation therapeutic target in breast cancer.

## Discussion

**Fig. 9:**
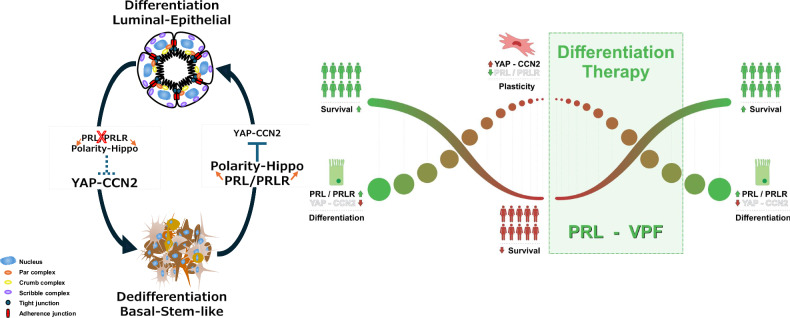
PRL/PRLR antagonism of YAP-CCN2 oncogenic pathway promotes differentiation and patient outcome in breast cancer and potential differentiation therapy outlook. In breast cancer luminal-to-basal dedifferentiation and cellular plasticity drives tumor heterogeneity and is associated with aggressive tumorigenic features and poor patient survival. PRL/PRLR signaling regulates and cooperates with polarity protein complexes and hippo pathway suppressing the oncogenic YAP-CCN2 pathway. This signaling network mediates acinar morphogenesis, protects the integrity of cell-cell junctions, promotes a differentiated luminal phenotype and extends patient survival. Thus, PRL and inhibitors of YAP-CCN2 pathway such as VPF exemplify differentiation therapeutic targets in breast cancer by targeting tumor cellular plasticity re-establishing luminal-epithelial differentiation, suppressing aggressive tumor phenotype, and leading to improved patient survival outcome.

The differentiation state of tumors has long been recognized as a key clinical marker in predicting tumor behavior, progression and metastasis. Molecular mechanisms and regulators promoting tumor cellular differentiation are proposed to reprogram cancer cells into a benign phenotype and are emerging as viable therapeutic avenues. In this study, we demonstrate that, in mammary and breast cancer cells, PRL/PRLR activates the apical-basal polarity cues and the Hippo signaling pathway, resulting in the suppression of the YAP-CCN2 oncogenic pathway that is critical in driving a differentiation program inducing/maintaining acinar morphogenesis, cell–cell junctions, limiting luminal-basal plasticity, and suppressing stemness. Together, our study emphasises the differentiation and anti-tumorigenic role of PRL in breast cancer by blocking the YAP-CCN2 oncogenic pathway providing prognostic and therapeutic strategies in breast cancer with also potential impact in other cancer types. Thus, promoting PRL/PRLR pathway and/or blocking YAP-CCN2 can be exploited as differentiation therapeutic targets in breast cancer (Fig. 9).

In the breast, epithelium apical-basal polarity organization is thought to create a barrier to breast tumorigenesis, and deregulation and/or loss of epithelial polarity is a feature of tumor progression and invasive phenotype [65]. Postnatal development of the mammary gland, especially during the pregnancy/lactation cycle, is accentuated by dramatic growth and morphological/architectural remodeling process termed alveologenesis, which supports milk production and lactation. During the pregnancy/lactation cycle, the mammary gland transforms from a ductal structure into a fully differentiated alveolar milk-secreting organ by the end of pregnancy and PRL/PRLR pathway is indispensable for this morphogenic programming [40, 41, 66]. Here, we showed PRL mediates structural and functional differentiation of the mammary epithelial cells by regulating the subcellular localization of the Par, Crumb, and Scribble core polarity protein complexes and restricts their apical and basal/lateral distribution to their respective domains thus highlighting the essential role of PRL in mammary differentiation. Within breast cancer setting using different breast cancer cell models, our results importantly highlighted that PRL’s ability to regulate acinar morphogenesis and A/B polarity can be extended to HR+ positive breast cancer cells. As well, PRL’s differentiation effects were also observed in the mesenchymal-claudin-low TNBC MDA-MB-231 cells as PRL treatment of these cells induced an epithelial-like phenotype typified by the formation of cellular aggregation/compaction marking the early events of establishment of the epithelial phenotype. Finally, the clinical impact of PRL pathway and polarity proteins in relation to breast cancer patient outcome was confirmed as the expression of PRLR positively correlates with most core polarity proteins and a gene signature of PRLR together with the highest correlated polarity genes PATJ, PALS1 and Dlg1 showed significant prognostic value, correlating with better OS and RFS in large breast cancer datasets. In summary, these results expand on the differentiation effects of PRL in mammary and breast cancer cell re-differentiation and its positive impact on patient survival outcome.

Reprogramming and lineage plasticity/conversion is a hallmark of cancer [1, 2]. In breast cancer, transition from a luminal to a basal-stem like cell identity promotes disease progression, aggressiveness, heterogeneity and resistance to therapy [4]. Therefore, characterizing mechanisms driving this luminal-basal conversion is of high relevance in decoding tumorigenesis and uncovering new anti-cancer therapeutic targets. Recent work characterizing the breast epithelium has confirmed that it is mainly divided into three major clusters: basal cells, mostly characterized by expression of KRT14, KRT17, KRT5, and ACTA2; luminal progenitors, enriched in the expression of ALDH1A3, KIT, CD24, PIGR, and Elf5; and mature luminal cells, also designated as luminal hormone responsive cells enriched in PRLR expression as well as KRT18, KRT8, ESR1, PGR, and FOXA1 [67–72]. PRL/PRLR signaling is known to regulate the mammary stem-cell hierarchy, deriving terminal differentiation [43]. Similarly, in breast cancer, PRL/PRLR signaling has been shown to suppress breast cancer stem cell populations [44]. Importantly, blocking PRLR expression in HR+ breast cancer MCF7 cells resulted in luminal-basal conversion with increased expression of mesenchymal-stem-like, tumorigenic and metastatic features as well as resistance to therapy [51]. While PRL has been shown to regulate Hippo pathway leading to pigeon crop epithelial cell proliferation [73], our study showed that PRL/PRLR/Jak2 pathway results in Hippo pathway activation and nuclear exclusion of YAP in mammary epithelial and breast cancer cells. Indeed, our study also indicates that the luminal-basal conversion upon loss of PRLR expression is linked to the inactivation of the Hippo signaling pathway, promoting the oncogenic YAP-CCN2 pathway. Indeed, blocking YAP with the chemical inhibitor VPF in MCF7/PRLR-KO cells reduced CCN2 levels and re-established proper cell polarity, tight and adherence junctions as well as luminal phenotype. Interestingly, YAP overexpression converted luminal differentiated mammary epithelial cells to a mammary-stem cell-like state MaSC-like state [74]. As well, inactivation of LATS1/2 was found to drive luminal-basal plasticity and initiate basal-like mammary carcinomas [75]. Moreover, YAP, CCN2 (CTGF) and CCN1 (Cyr61) were also found to be overexpressed in tamoxifen-resistant breast cancer and induced transcriptional repression of ERα [76]. Similarly, a variant of MCF7 that has undergone EMT, designated as MCF7-EMT, was also shown to express high levels of CCN2 [29]. Altogether, these results highlight that in mammary and breast cancer cells, the pro-differentiation effects of PRL/PRLR are closely linked to Hippo pathway promoting cell polarity and luminal linage differentiation.

Previously a pro-tumorigenic role for PRL in BC has been proposed [77, 78]. Therefore, targeting PRL/PRLR pathway as a therapeutic avenue in breast cancer has been a topic of interest for decades. Indeed, efforts have been directed to block PRL as a therapeutic avenue in breast cancer, including the generation of PRL antagonists (Del1-9-G129R-hPRL, G129R-Prl) [79, 80] and PRLR antagonist (LFA102) [81, 82]. However, none of these agents have resulted in FDA approval to date [83]. Notably, our study provides evidence that inducing and/or maintaining PRL/PRLR signaling is critical in restricting and suppressing breast tumorigenesis and promoting its differentiation function may hold therapeutic value in breast cancer. Furthermore, our study also points to the therapeutic potential of combination therapeutic strategies exploiting the clinically relevant antagonistic relation between PRL/PRLR and the YAP-CCN2 axis discovered here. Indeed, pharmacological inhibitors of YAP are currently being evaluated in a phase I clinical trial in tumors bearing mutations in Hippo pathway components or YAP/TAZ gene fusions (NCT04857372) and humanized anti-CCN2 antibodies are currently in clinical investigation (ClinicalTrials.gov, NCT01890265) in pancreatic cancer and (Phase III, ClinicalTrials.gov, NCT03955146) fibrotic diseases [84–87]. Our study highlights a potential combination therapeutic modality based on PRL and YAP inhibitors or PRL and anti-CCN2 antibodies in breast cancer. These treatment approaches could have therapeutic value in the clinically challenging so far incurable metastatic breast cancers that usually are of high grade (poorly differentiated) showing luminal/basal conversion/plasticity through re-differentiation restricting their aggressive and proliferative phenotype.

## Supplementary information

Supplemental Material

## Data Availability

Four RNA libraries from MCF7/SCR and 4 libraries from MCF7/PRLR-KO cells were sequenced at McGill Genome Centre with 100 bp stranded paired-end reads, and the sequencing data has been deposited in the NCBI website, www.ncbi.nlm.nih.gov/geo (accession no. PRJNA1123250). Survival curves were gathered by Kaplan–Meier plotter database (http://kmplot.com/analysis/index.php?p=service&cancer=breast). The expression profile and co-expression analysis of CCN2 and the polarity complex genes in BC were extracted from the Sweden Cancerome Analysis Network-Breast (SCAN-B) database (GSE202203). Additionally, data from the microdissected breast epithelium (GSE141828) of healthy women and the Molecular Taxonomy of BC International Consortium (METABRIC) database were downloaded and processed using Python scripts. Boxplot comparative view of PRLR, CCN2 and polarity complex gene expression was collected from SCAN-B dataset. A gene-gene interaction network for the CCN2 gene was constructed using GeneMania and visualized through Cytoscape. The graphical representation illustrates Gene Ontology and pathway enrichment analyses performed using Enrichr on the top 20 enriched genes associated with the CCN2 gene. GO:BP and WIKI were used to represent the biological processes and pathways. The bar graph depicts the co-expression of Hippo pathway genes (hsa04390) from the KEGG database with the PRLR gene. A STRING Protein-Protein Interaction (PPI) [88] network was generated for the 18 genes correlated with PRLR from the KEGG Hippo pathway. Images showing single confocal slices were adjusted for brightness with Adobe Photoshop CS6 and composite images with scale bars were made with Adobe Illustrator CS6.

## References

[1] Bhat GR, Sethi I, Sadida HQ, Rah B, Mir R, Algehainy N, et al. Cancer cell plasticity: from cellular, molecular, and genetic mechanisms to tumor heterogeneity and drug resistance. Cancer Metastasis Rev. 2024;43:197–228. 38329598 10.1007/s10555-024-10172-z PMC11016008

[2] Hanahan D. Hallmarks of cancer: new dimensions. Cancer Discov. 2022;12:31–46. 35022204 10.1158/2159-8290.CD-21-1059

[3] Mehta A, Stanger BZ. Lineage plasticity: the new cancer hallmark on the block. Cancer Res. 2024;84:184–91. 37963209 10.1158/0008-5472.CAN-23-1067 PMC10841583

[4] Gupta PB, Pastushenko I, Skibinski A, Blanpain C, Kuperwasser C. Phenotypic plasticity: driver of cancer initiation, progression, and therapy resistance. Cell Stem Cell. 2019;24:65–78. 30554963 10.1016/j.stem.2018.11.011 PMC7297507

[5] Xu WP, Zhang X, Xie WF. Differentiation therapy for solid tumors. J Dig Dis. 2014;15:159–65. 24373518 10.1111/1751-2980.12122

[6] Leszczyniecka M, Roberts T, Dent P, Grant S, Fisher PB. Differentiation therapy of human cancer: basic science and clinical applications. Pharmacol Ther. 2001;90:105–56. 11578655 10.1016/s0163-7258(01)00132-2

[7] de The H. Differentiation therapy revisited. Nat Rev Cancer. 2018;18:117–27. 29192213 10.1038/nrc.2017.103

[8] Cruz FD, Matushansky I. Solid tumor differentiation therapy - is it possible?. Oncotarget. 2012;3:559–67. 22643847 10.18632/oncotarget.512 PMC3388185

[9] Curtis C, Shah SP, Chin SF, Turashvili G, Rueda OM, Dunning MJ, et al. The genomic and transcriptomic architecture of 2,000 breast tumours reveals novel subgroups. Nature. 2012;486:346–52. 22522925 10.1038/nature10983 PMC3440846

[10] Sørlie T. Molecular portraits of breast cancer: tumour subtypes as distinct disease entities. European J Cancer. 2004;40:2667–75. 15571950 10.1016/j.ejca.2004.08.021

[11] Prat A, Perou CM. Deconstructing the molecular portraits of breast cancer. Mol Oncol. 2011;5:5–23. 21147047 10.1016/j.molonc.2010.11.003 PMC5528267

[12] Jacobs AT, Martinez Castaneda-Cruz D, Rose MM, Connelly L. Targeted therapy for breast cancer: An overview of drug classes and outcomes. Biochem Pharmacol. 2022;204:115209. 35973582 10.1016/j.bcp.2022.115209

[13] Cortesi L, Rugo HS, Jackisch C. An overview of PARP inhibitors for the treatment of breast cancer. Target Oncol. 2021;16:255–82. 33710534 10.1007/s11523-021-00796-4 PMC8105250

[14] Debien V, De Caluwe A, Wang X, Piccart-Gebhart M, Tuohy VK, Romano E, et al. Immunotherapy in breast cancer: an overview of current strategies and perspectives. NPJ Breast Cancer. 2023;9:7. 36781869 10.1038/s41523-023-00508-3 PMC9925769

[15] Peglion F, Etienne-Manneville S. Cell polarity changes in cancer initiation and progression. J Cell Biol. 2024;223:e202308069. 38091012 10.1083/jcb.202308069 PMC10720656

[16] Rodriguez-Boulan E, Macara IG. Organization and execution of the epithelial polarity programme. Nat Rev Mol Cell Biol. 2014;15:225–42. 24651541 10.1038/nrm3775 PMC4211427

[17] Chatterjee SJ, McCaffrey L. Emerging role of cell polarity proteins in breast cancer progression and metastasis. Breast Cancer (Dove Med Press). 2014;6:15–27. 24648766 10.2147/BCTT.S43764 PMC3929326

[18] Mao X, Liang Z, Chibhabha F, Ou W, Li N, Xu P, et al. Clinico-radiological features and next generation sequencing of pulmonary epithelioid hemangioendothelioma: a case report and review of literature. Thorac Cancer. 2017;8:687–92. 28777494 10.1111/1759-7714.12474 PMC5668507

[19] Yang CC, Graves HK, Moya IM, Tao C, Hamaratoglu F, Gladden AB, et al. Differential regulation of the Hippo pathway by adherens junctions and apical-basal cell polarity modules. Proc Natl Acad Sci USA. 2015;112:1785–90. 25624491 10.1073/pnas.1420850112 PMC4330745

[20] Zhou PJ, Wang X, An N, Wei L, Zhang L, Huang X, et al. Loss of Par3 promotes prostatic tumorigenesis by enhancing cell growth and changing cell division modes. Oncogene. 2019;38:2192–205. 30467379 10.1038/s41388-018-0580-x

[21] Yu FX, Guan KL. The Hippo pathway: regulators and regulations. Genes Dev. 2013;27:355–71. 23431053 10.1101/gad.210773.112 PMC3589553

[22] Meng Z, Moroishi T, Guan KL. Mechanisms of Hippo pathway regulation. Genes Dev. 2016;30:1–17. 26728553 10.1101/gad.274027.115 PMC4701972

[23] Zanconato F, Cordenonsi M, Piccolo S. YAP/TAZ at the roots of cancer. Cancer Cell. 2016;29:783–803. 27300434 10.1016/j.ccell.2016.05.005 PMC6186419

[24] Brigstock DR, Goldschmeding R, Katsube KI, Lam SC, Lau LF, Lyons K, et al. Proposal for a unified CCN nomenclature. Mol Pathol. 2003;56:127–8. 12665631 10.1136/mp.56.2.127 PMC1187305

[25] Leask A. Conjunction junction, what’s the function? CCN proteins as targets in fibrosis and cancers. Am J Physiol Cell Physiol. 2020;318:C1046–C54. 32130070 10.1152/ajpcell.00028.2020 PMC7311738

[26] Sun C, Zhang H, Liu X. Emerging role of CCN family proteins in fibrosis. J Cell Physiol. 2021;236:4195–206. 33222181 10.1002/jcp.30171

[27] Kim H, Son S, Ko Y, Shin I. CTGF regulates cell proliferation, migration, and glucose metabolism through activation of FAK signaling in triple-negative breast cancer. Oncogene. 2021;40:2667–81. 33692467 10.1038/s41388-021-01731-7

[28] Xie D, Nakachi K, Wang H, Elashoff R, Koeffler HP. Elevated levels of connective tissue growth factor, WISP-1, and CYR61 in primary breast cancers associated with more advanced features. Cancer Res. 2001;61:8917–23. 11751417

[29] Hellinger JW, Schomel F, Buse JV, Lenz C, Bauerschmitz G, Emons G, et al. Identification of drivers of breast cancer invasion by secretome analysis: insight into CTGF signaling. Sci Rep. 2020;10:17889. 33087801 10.1038/s41598-020-74838-8 PMC7578015

[30] Moran-Jones K, Gloss BS, Murali R, Chang DK, Colvin EK, Jones MD, et al. Connective tissue growth factor as a novel therapeutic target in high grade serous ovarian cancer. Oncotarget. 2015;6:44551–62. 26575166 10.18632/oncotarget.6082 PMC4792575

[31] Finger EC, Cheng CF, Williams TR, Rankin EB, Bedogni B, Tachiki L, et al. CTGF is a therapeutic target for metastatic melanoma. Oncogene. 2014;33:1093–100. 23435419 10.1038/onc.2013.47 PMC3965577

[32] Ren W, Sun X, Wang K, Feng H, Liu Y, Fei C, et al. BMP9 inhibits the bone metastasis of breast cancer cells by downregulating CCN2 (connective tissue growth factor, CTGF) expression. Mol Biol Rep. 2014;41:1373–83. 24413988 10.1007/s11033-013-2982-8

[33] Deng YZ, Chen PP, Wang Y, Yin D, Koeffler HP, Li B, et al. Connective tissue growth factor is overexpressed in esophageal squamous cell carcinoma and promotes tumorigenicity through beta-catenin-T-cell factor/Lef signaling. J Biol Chem. 2007;282:36571–81. 17951630 10.1074/jbc.M704141200

[34] Shimo T, Kubota S, Yoshioka N, Ibaragi S, Isowa S, Eguchi T, et al. Pathogenic role of connective tissue growth factor (CTGF/CCN2) in osteolytic metastasis of breast cancer. J Bone Min Res. 2006;21:1045–59. 10.1359/jbmr.060416 16813525

[35] Wu Z, Su J, Li FL, Chen T, Mayner J, Engler A, et al. YAP silencing by RB1 mutation is essential for small-cell lung cancer metastasis. Nat Commun. 2023;14:5916. 37739954 10.1038/s41467-023-41585-z PMC10516997

[36] Sadri F, Hosseini SF, Rezaei Z, Fereidouni M. Hippo-YAP/TAZ signaling in breast cancer: reciprocal regulation of microRNAs and implications in precision medicine. Genes Dis. 2024;11:760–71. 37692482 10.1016/j.gendis.2023.01.017 PMC10491881

[37] Dey A, Varelas X, Guan KL. Targeting the Hippo pathway in cancer, fibrosis, wound healing and regenerative medicine. Nat Rev Drug Discov. 2020;19:480–94. 32555376 10.1038/s41573-020-0070-z PMC7880238

[38] Piccolo S, Panciera T, Contessotto P, Cordenonsi M. YAP/TAZ as master regulators in cancer: modulation, function and therapeutic approaches. Nat Cancer. 2023;4:9–26. 36564601 10.1038/s43018-022-00473-z PMC7614914

[39] Dai M, Yan G, Wang N, Daliah G, Edick AM, Poulet S, et al. In vivo genome-wide CRISPR screen reveals breast cancer vulnerabilities and synergistic mTOR/Hippo targeted combination therapy. Nat Commun. 2021;12:3055. 34031411 10.1038/s41467-021-23316-4 PMC8144221

[40] Anderson SM, Rudolph MC, McManaman JL, Neville MC. Key stages in mammary gland development. Secretory activation in the mammary gland: it’s not just about milk protein synthesis!. Breast Cancer Res. 2007;9:204. 17338830 10.1186/bcr1653 PMC1851396

[41] Slepicka PF, Somasundara AVH, Dos Santos CO. The molecular basis of mammary gland development and epithelial differentiation. Semin Cell Dev Biol. 2021;114:93–112. 33082117 10.1016/j.semcdb.2020.09.014 PMC8052380

[42] Hannan FM, Elajnaf T, Vandenberg LN, Kennedy SH, Thakker RV. Hormonal regulation of mammary gland development and lactation. Nat Rev Endocrinol. 2023;19:46–61. 36192506 10.1038/s41574-022-00742-y

[43] Liu F, Pawliwec A, Feng Z, Yasruel Z, Lebrun JJ, Ali S. Prolactin/Jak2 directs apical/basal polarization and luminal linage maturation of mammary epithelial cells through regulation of the Erk1/2 pathway. Stem Cell Res. 2015;15:376–83. 26318719 10.1016/j.scr.2015.08.001

[44] Ali S, Hamam D, Liu X, Lebrun JJ. Terminal differentiation and anti-tumorigenic effects of prolactin in breast cancer. Front Endocrinol. 2022;13:993570. 10.3389/fendo.2022.993570 PMC9499354 36157462

[45] Kalinina TS, Kononchuk VV, Sidorov SV, Gulyaeva LF. Analysis of prolactin receptor expression in breast cancer subtypes. Biomed Khim. 2020;66:89–94. 32116231 10.18097/PBMC20206601089

[46] Hachim IY, Shams A, Lebrun JJ, Ali S. A favorable role of prolactin in human breast cancer reveals novel pathway-based gene signatures indicative of tumor differentiation and favorable patient outcome. Hum Pathol. 2016;53:142–52. 26980025 10.1016/j.humpath.2016.02.010

[47] Nitze LM, Galsgaard ED, Din N, Lund VL, Rasmussen BB, Berchtold MW, et al. Reevaluation of the proposed autocrine proliferative function of prolactin in breast cancer. Breast Cancer Res Treat. 2013;142:31–44. 24146212 10.1007/s10549-013-2731-7 PMC3825490

[48] Hachim IY, Hachim MY, Lopez VM, Lebrun JJ, Ali S. Prolactin receptor expression is an independent favorable prognostic marker in human breast cancer. Appl Immunohistochem Mol Morphol. 2016;24:238–45. 26317306 10.1097/PAI.0000000000000178

[49] Lopez-Ozuna VM, Hachim IY, Hachim MY, Lebrun JJ, Ali S. Prolactin modulates TNBC aggressive phenotype limiting tumorigenesis. Endocr Relat Cancer. 2019;26:321–37. 30640712 10.1530/ERC-18-0523

[50] Lopez-Ozuna VM, Hachim IY, Hachim MY, Lebrun JJ, Ali S. Prolactin pro-differentiation pathway in triple negative breast cancer: impact on prognosis and potential therapy. Sci Rep. 2016;6:30934. 27480353 10.1038/srep30934 PMC4969612

[51] Shams A, Binothman N, Boudreault J, Wang N, Shams F, Hamam D, et al. Prolactin receptor-driven combined luminal and epithelial differentiation in breast cancer restricts plasticity, stemness, tumorigenesis and metastasis. Oncogenesis. 2021;10:10. 33446633 10.1038/s41389-020-00297-5 PMC7809050

[52] Ewels P, Magnusson M, Lundin S, Kaller M. MultiQC: summarize analysis results for multiple tools and samples in a single report. Bioinformatics. 2016;32:3047–8. 27312411 10.1093/bioinformatics/btw354 PMC5039924

[53] Dobin A, Davis CA, Schlesinger F, Drenkow J, Zaleski C, Jha S, et al. STAR: ultrafast universal RNA-seq aligner. Bioinformatics. 2013;29:15–21. 23104886 10.1093/bioinformatics/bts635 PMC3530905

[54] Anders S, Pyl PT, Huber W. HTSeq-a Python framework to work with high-throughput sequencing data. Bioinformatics. 2015;31:166–9. 25260700 10.1093/bioinformatics/btu638 PMC4287950

[55] Love MI, Huber W, Anders S. Moderated estimation of fold change and dispersion for RNA-seq data with DESeq2. Genome Biol. 2014;15:550. 25516281 10.1186/s13059-014-0550-8 PMC4302049

[56] Xie Z, Bailey A, Kuleshov MV, Clarke DJB, Evangelista JE, Jenkins SL, et al. Gene Set Knowledge Discovery with Enrichr. Curr Protoc. 2021;1:e90. 33780170 10.1002/cpz1.90 PMC8152575

[57] Franz M, Rodriguez H, Lopes C, Zuberi K, Montojo J, Bader GD, et al. GeneMANIA update 2018. Nucleic Acids Res. 2018;46:W60–W4. 29912392 10.1093/nar/gky311 PMC6030815

[58] Shannon P, Markiel A, Ozier O, Baliga NS, Wang JT, Ramage D, et al. Cytoscape: a software environment for integrated models of biomolecular interaction networks. Genome Res. 2003;13:2498–504. 14597658 10.1101/gr.1239303 PMC403769

[59] Dalal H, Dahlgren M, Gladchuk S, Brueffer C, Gruvberger-Saal SK, Saal LH. Clinical associations of ESR2 (estrogen receptor beta) expression across thousands of primary breast tumors. Sci Rep. 2022;12:4696. 35304506 10.1038/s41598-022-08210-3 PMC8933558

[60] Marino N, German R, Rao X, Simpson E, Liu S, Wan J, et al. Upregulation of lipid metabolism genes in the breast prior to cancer diagnosis. NPJ Breast Cancer. 2020;6:50. 33083529 10.1038/s41523-020-00191-8 PMC7538898

[61] Kanehisa M. Goto S. KEGG: kyoto encyclopedia of genes and genomes. Nucleic Acids Res. 2000;28:27–30. 10592173 10.1093/nar/28.1.27 PMC102409

[62] Raina P, Guinea R, Chatsirisupachai K, Lopes I, Farooq Z, Guinea C, et al. GeneFriends: gene co-expression databases and tools for humans and model organisms. Nucleic Acids Res. 2023;51:D145–D58. 36454018 10.1093/nar/gkac1031 PMC9825523

[63] Haines E, Minoo P, Feng Z, Resalatpanah N, Nie XM, Campiglio M, et al. Tyrosine phosphorylation of Grb2: role in prolactin/epidermal growth factor cross talk in mammary epithelial cell growth and differentiation. Mol Cell Biol. 2009;29:2505–20. 19273609 10.1128/MCB.00034-09 PMC2682022

[64] Gyorffy B, Lanczky A, Eklund AC, Denkert C, Budczies J, Li Q, et al. An online survival analysis tool to rapidly assess the effect of 22,277 genes on breast cancer prognosis using microarray data of 1,809 patients. Breast Cancer Res Treat. 2010;123:725–31. 20020197 10.1007/s10549-009-0674-9

[65] Catterall R, Lelarge V, McCaffrey L. Genetic alterations of epithelial polarity genes are associated with loss of polarity in invasive breast cancer. Int J Cancer. 2020;146:1578–91. 31577845 10.1002/ijc.32691

[66] Ormandy CJ, Naylor M, Harris J, Robertson F, Horseman ND, Lindeman GJ, et al. Investigation of the transcriptional changes underlying functional defects in the mammary glands of prolactin receptor knockout mice. Recent Prog Horm Res. 2003;58:297–323. 12795425 10.1210/rp.58.1.297

[67] Pal B, Chen Y, Vaillant F, Jamieson P, Gordon L, Rios AC, et al. Construction of developmental lineage relationships in the mouse mammary gland by single-cell RNA profiling. Nat Commun. 2017;8:1627. 29158510 10.1038/s41467-017-01560-x PMC5696379

[68] Bach K, Pensa S, Grzelak M, Hadfield J, Adams DJ, Marioni JC, et al. Differentiation dynamics of mammary epithelial cells revealed by single-cell RNA sequencing. Nat Commun. 2017;8. 2128. 29225342 10.1038/s41467-017-02001-5 PMC5723634

[69] Gray GK, Li CM, Rosenbluth JM, Selfors LM, Girnius N, Lin JR, et al. A human breast atlas integrating single-cell proteomics and transcriptomics. Dev Cell. 2022;57:1400–20.e7. 35617956 10.1016/j.devcel.2022.05.003 PMC9202341

[70] Pal B, Chen Y, Vaillant F, Capaldo BD, Joyce R, Song X, et al. A single-cell RNA expression atlas of normal, preneoplastic and tumorigenic states in the human breast. EMBO J. 2021;40:e107333. 33950524 10.15252/embj.2020107333 PMC8167363

[71] Giraddi RR, Chung CY, Heinz RE, Balcioglu O, Novotny M, Trejo CL, et al. Single-Cell Transcriptomes Distinguish Stem Cell State Changes and Lineage Specification Programs in Early Mammary Gland Development. Cell Rep. 2018;24:1653–66.e7. 30089273 10.1016/j.celrep.2018.07.025 PMC6301014

[72] Kumar T, Nee K, Wei R, He S, Nguyen QH, Bai S, et al. A spatially resolved single-cell genomic atlas of the adult human breast. Nature. 2023;620:181–91. 37380767 10.1038/s41586-023-06252-9 PMC11443819

[73] Zhu J, Teng X, Wang L, Zheng M, Meng Y, Liu T, et al. Prolactin promotes crop epithelial proliferation of domestic pigeons (Columba livia) through the Hippo signaling pathway. J Anim Sci. 2023;101:skad312. 10.1093/jas/skad312 PMC10576522 37721785

[74] Panciera T, Azzolin L, Fujimura A, Di Biagio D, Frasson C, Bresolin S, et al. Induction of expandable tissue-specific stem/progenitor cells through transient expression of YAP/TAZ. Cell Stem Cell. 2016;19:725–37. 27641305 10.1016/j.stem.2016.08.009 PMC5145813

[75] Kern JG, Tilston-Lunel AM, Federico A, Ning B, Mueller A, Peppler GB, et al. Inactivation of LATS1/2 drives luminal-basal plasticity to initiate basal-like mammary carcinomas. Nat Commun. 2022;13:7198. 36443313 10.1038/s41467-022-34864-8 PMC9705439

[76] Kim H, Son S, Ko Y, Lee JE, Kim S, Shin I. YAP, CTGF and Cyr61 are overexpressed in tamoxifen-resistant breast cancer and induce transcriptional repression of ERalpha. J Cell Sci. 2021;134:jcs256503. 10.1242/jcs.256503 34096606

[77] Clevenger CV, Chang WP, Ngo W, Pasha TL, Montone KT, Tomaszewski JE. Expression of prolactin and prolactin receptor in human breast carcinoma. Evidence for an autocrine/paracrine loop. Am J Pathol. 1995;146:695–705. 7534043 PMC1869171

[78] Maus MV, Reilly SC, Clevenger CV. Prolactin as a chemoattractant for human breast carcinoma. Endocrinology. 1999;140:5447–50. 10537179 10.1210/endo.140.11.7245

[79] Goffin V, Bernichtein S, Touraine P, Kelly PA. Development and potential clinical uses of human prolactin receptor antagonists. Endocr Rev. 2005;26:400–22. 15814850 10.1210/er.2004-0016

[80] Howell SJ, Anderson E, Hunter T, Farnie G, Clarke RB. Prolactin receptor antagonism reduces the clonogenic capacity of breast cancer cells and potentiates doxorubicin and paclitaxel cytotoxicity. Breast Cancer Res. 2008;10:R68. 18681966 10.1186/bcr2129 PMC2575541

[81] Damiano JS, Wasserman E. Molecular pathways: blockade of the PRLR signaling pathway as a novel antihormonal approach for the treatment of breast and prostate cancer. Clin Cancer Res. 2013;19:1644–50. 23515410 10.1158/1078-0432.CCR-12-0138

[82] Agarwal N, Machiels JP, Suarez C, Lewis N, Higgins M, Wisinski K, et al. Phase I study of the prolactin receptor antagonist LFA102 in metastatic breast and castration-resistant prostate cancer. Oncologist. 2016;21:535–6. 27091421 10.1634/theoncologist.2015-0502 PMC4861370

[83] Standing D, Dandawate P, Anant S. Prolactin receptor signaling: A novel target for cancer treatment - exploring anti-PRLR signaling strategies. Front Endocrinol. 2022;13:1112987. 10.3389/fendo.2022.1112987 PMC9880166 36714582

[84] Aikawa T, Gunn J, Spong SM, Klaus SJ, Korc M. Connective tissue growth factor-specific antibody attenuates tumor growth, metastasis, and angiogenesis in an orthotopic mouse model of pancreatic cancer. Mol Cancer Ther. 2006;5:1108–16. 16731742 10.1158/1535-7163.MCT-05-0516

[85] Dornhofer N, Spong S, Bennewith K, Salim A, Klaus S, Kambham N, et al. Connective tissue growth factor-specific monoclonal antibody therapy inhibits pancreatic tumor growth and metastasis. Cancer Res. 2006;66:5816–27. 16740721 10.1158/0008-5472.CAN-06-0081

[86] Neesse A, Frese KK, Bapiro TE, Nakagawa T, Sternlicht MD, Seeley TW, et al. CTGF antagonism with mAb FG-3019 enhances chemotherapy response without increasing drug delivery in murine ductal pancreas cancer. Proc Natl Acad Sci USA. 2013;110:12325–30. 23836645 10.1073/pnas.1300415110 PMC3725120

[87] Richeldi L, Fernandez Perez ER, Costabel U, Albera C, Lederer DJ, Flaherty KR, et al. Pamrevlumab, an anti-connective tissue growth factor therapy, for idiopathic pulmonary fibrosis (PRAISE): a phase 2, randomised, double-blind, placebo-controlled trial. Lancet Respir Med. 2020;8:25–33. 31575509 10.1016/S2213-2600(19)30262-0

[88] Szklarczyk D, Kirsch R, Koutrouli M, Nastou K, Mehryary F, Hachilif R, et al. The STRING database in 2023: protein-protein association networks and functional enrichment analyses for any sequenced genome of interest. Nucleic Acids Res. 2023;51:D638–D46. 36370105 10.1093/nar/gkac1000 PMC9825434

