# Supplementary material for: A novel clinically relevant antagonistic interplay between prolactin and oncogenic YAP-CCN2 pathways as a differentiation therapeutic target in breast cancer
Source: Cell Death Dis. 2025 Mar 29;16(1):221. doi: 10.1038/s41419-025-07547-7 (PMC11954952; doi:10.1038/s41419-025-07547-7)
Supplement: Supplementary file 1 — Supplemental Material [file 41419_2025_7547_MOESM1_ESM.pdf]

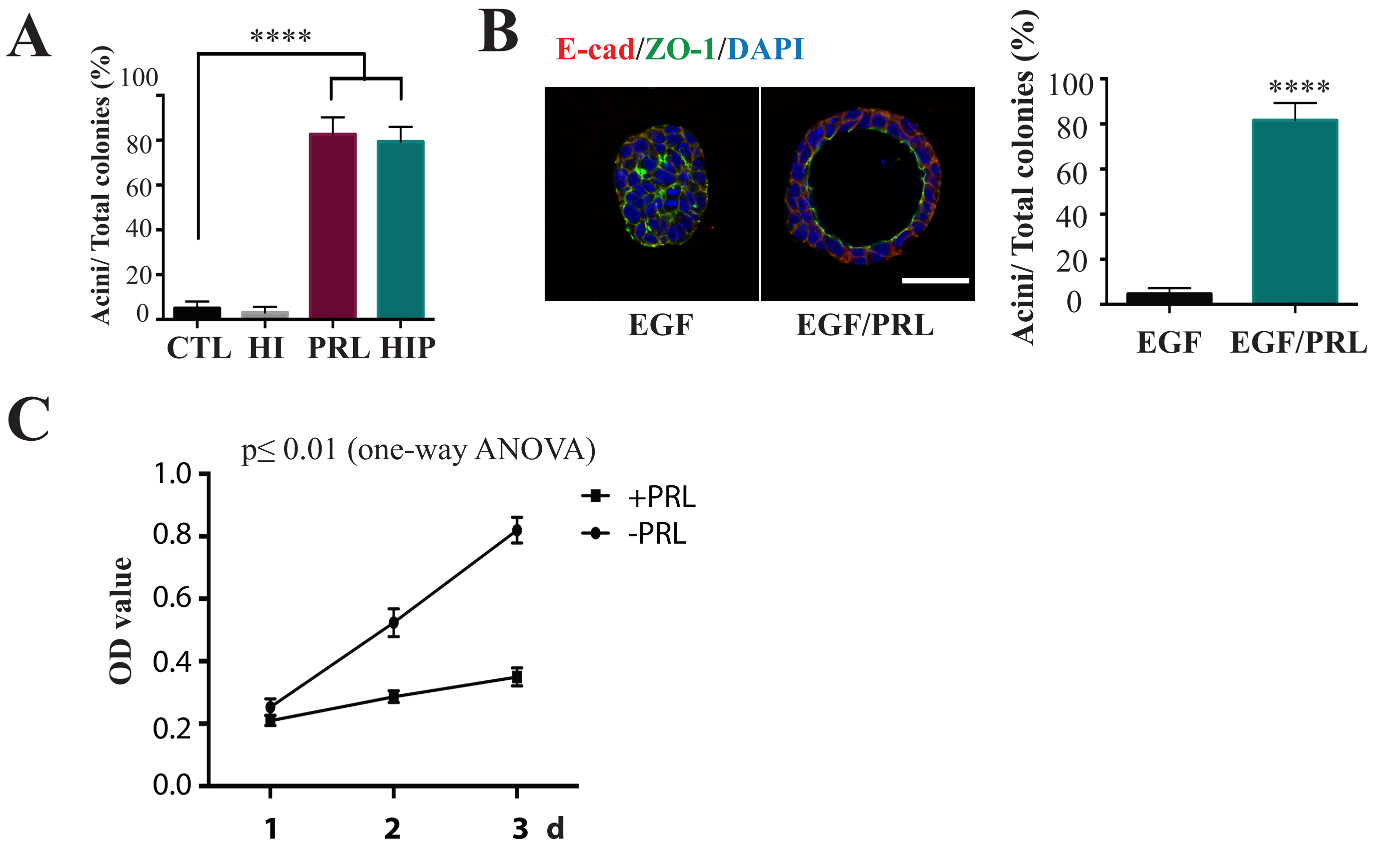

S1. PRL/EGF crosstalk in acinar morphogenesis and MEC proliferation.
A. MECs *ex-vivo* grown as 3D cultures on Matrigel gel under different treatment conditions (CTL, HI, PRL or HIP) for 3 days. The graph showed the quantification percentage of mammary acini in total colonies counted (more than 100 colonies per group). Graph showed mean ± SEM of triplicates of 3 independent experiments. ****$p < 0.0001$. The p-values were derived using ANOVA test. **B.** Left panel, MECs grown in 3D culture condition in the presence of either EGF alone or a combination of PRL and EGF. Cells were then co-stained with antibodies to E-cad (red) and ZO-1 (green). Scale bar, 20 µm. Right panel depicts the percentage of mammary acini in total colonies (more than 100 colonies were counted for each group). Graph showed mean ± SEM of 3 independent experiments. ****$p \leq 0.0001$ (unpaired two-tailed Student's t-test). **C.** MECs were grown in the presence of EGF ± PRL. MTT assays were performed for 1, 2 and 3 days. Results are expressed as mean ± SD of 3 independent experiments. $p \leq 0.01$ (one-way ANOVA).

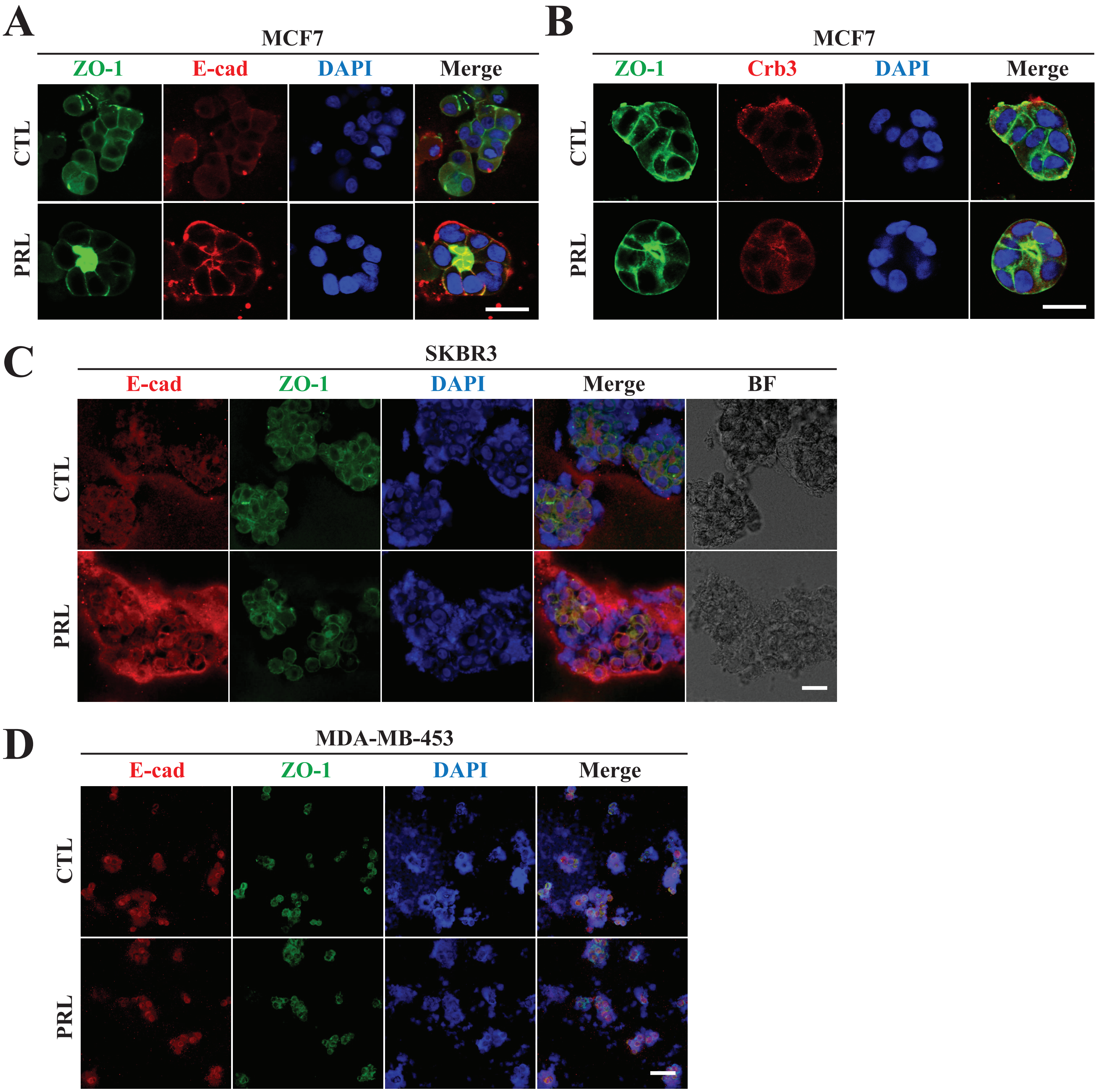

S2. PRL regulation of acinar morphogenesis in MCF7, SKBR3 and MDA-MB-453 human breast cancer cells.

Acinar morphogenesis in breast cancer cellular models MCF7 **(A & B)**, SKBR3 **(C)** and MDA-MB-453 **(D)**. Cells were grown in 3D culture conditions (Materials and Methods) without (CTL) or with PRL and co-stained with antibodies to E-cad (red), ZO-1 (green) or Crb3 (red), asa indicated on top. Nuclei were counter stained with DAPI (blue). Scale bar, 20 µm.

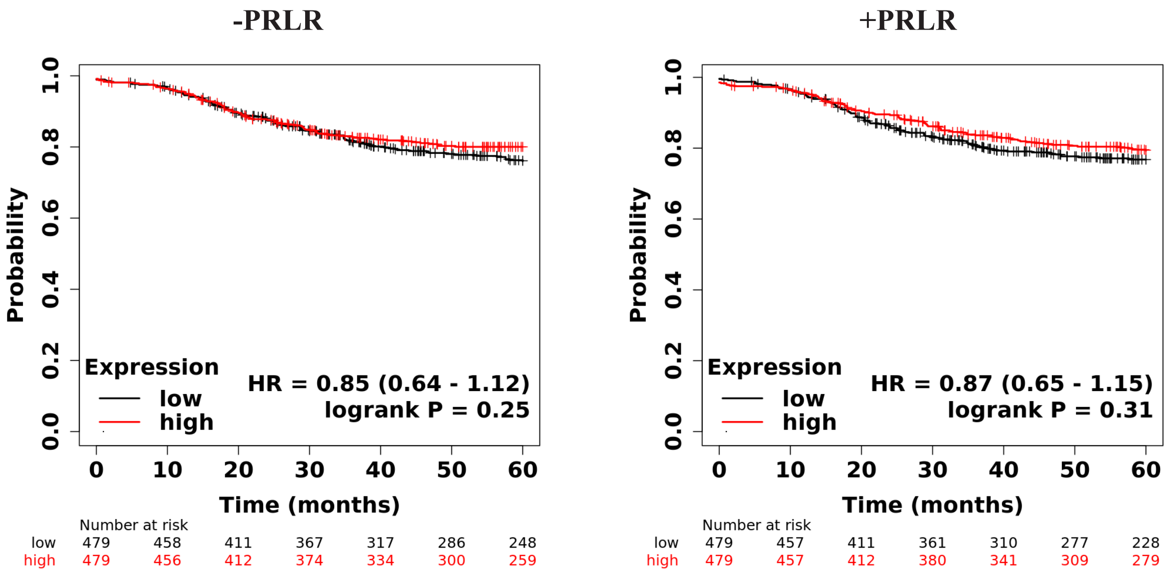

S3. Survival curves for the gene signatures PATJ, PALS1 and DLG1 without (left) or with (right) PRLR gene using Distant Metastasis-Free Survival (DMFS) as an endpoint in breast cancer patient's samples using the Kaplan-Meier plotter database.

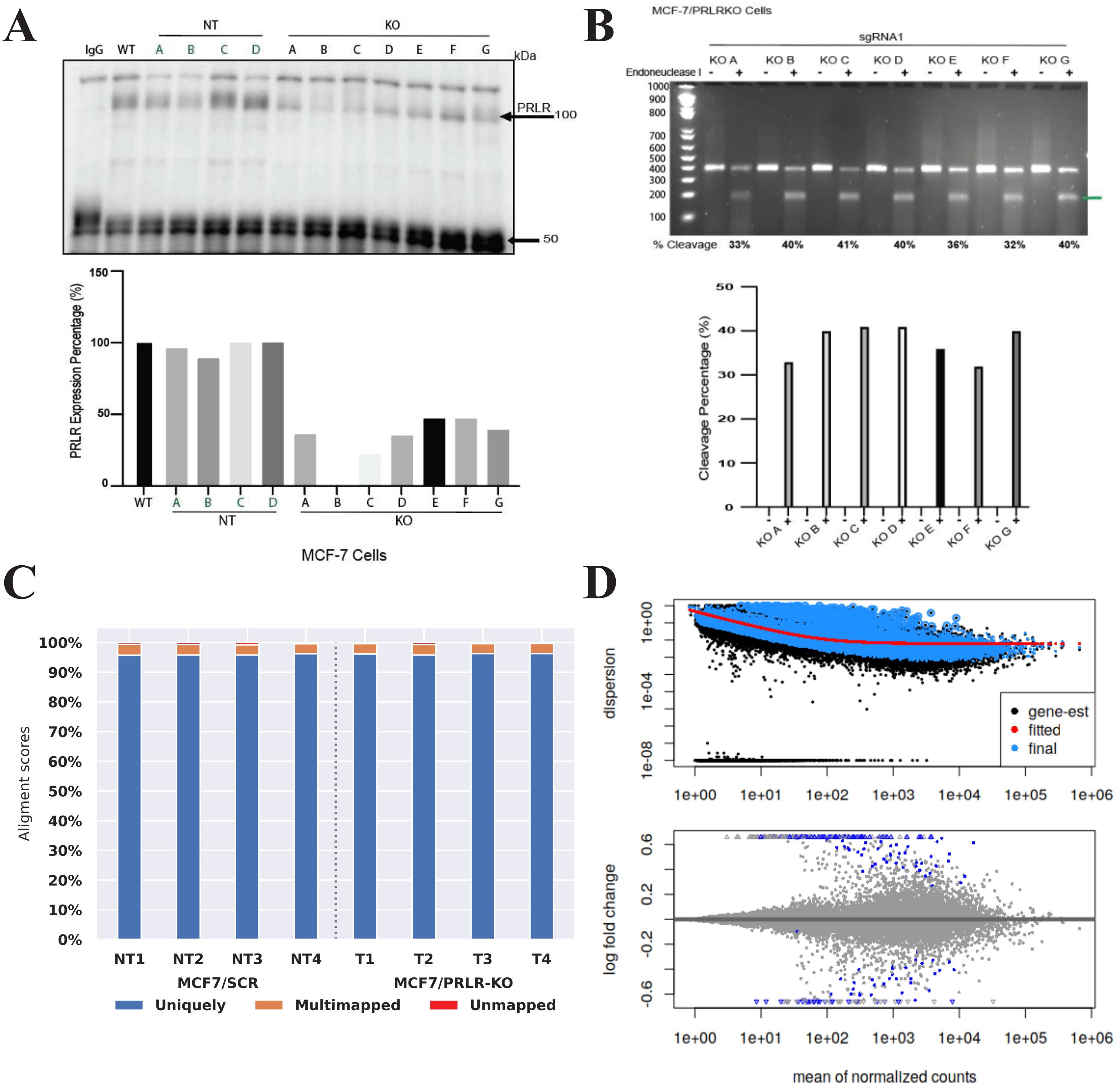

S4. Verification of CRISPR/Cas9 PRLR-KO in MCF7 cells and bulk RNA-sequencing database.

A. Western blots verification of CRISPR/Cas9 PRLR knockout biological replicates cell lines. MCF7 none-targeting (NT, A-D) cells and MCF7/PRLR-KO (A-G) cells were lysed and immunoprecipitations were performed using a rabbit polyclonal antibody against the PRLR (Sigma, SAB1401312) or control normal rabbit IgG. Western blotting was carried out using a mouse monoclonal antibody against the PRLR (Santa-Cruz, sc-377098). Quantitative analysis of PRLR expression normalized to the WT was shown below.

B. Agarose gel electrophoresis of nuclease-treated PCR products of MCF-7 knockout cells. A mixture containing KO A (lanes 1, 2), KO B (lanes 3, 4), KO C (lanes 5, 6), KO D (lanes 7, 8), KO E (lanes 9, 10), KO F (lanes 11, 12), & KO G (lanes 13, 14), was re-annealed and untreated or treated with endonuclease I. Green arrow indicates the expected cleavage bands. Densitometry was performed on all samples and quantification analysis of the cleavage percentage obtained in each sample is indicated.

C. Mapping statistics for SCR (NT) and PRLR-KO (T) RNA-seq samples. Data was collected from STAR alignment and each sample was plotted with its respective percentage of uniquely mapped, multi-mapped and unmapped reads.

D. Dispersion and MA plots were generated using the DESeq2 package. The dispersion plot illustrates the variation in gene expression between samples (Y-axis) across different average expression levels (X-axis). A fitted red line represents the typical variation trend. The MA plot displays the log2 fold change against the mean average expression for each gene. Altered gene expression levels between the two conditions are represented by blue dots.

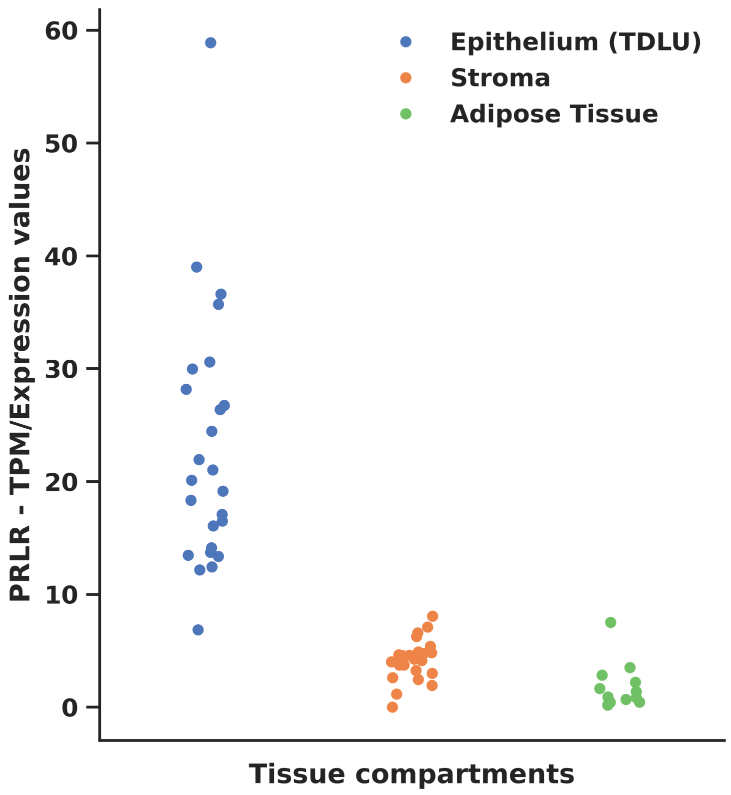

S5. PRLR expression profile from microdissected breast epithelium RNA libraries.
The scatter plot shows the expression profile of the PRLR gene in transcripts per million (TPM) across the epithelium (TDLU), stroma, and adipose tissue compartments of healthy human breast tissues (n=23).

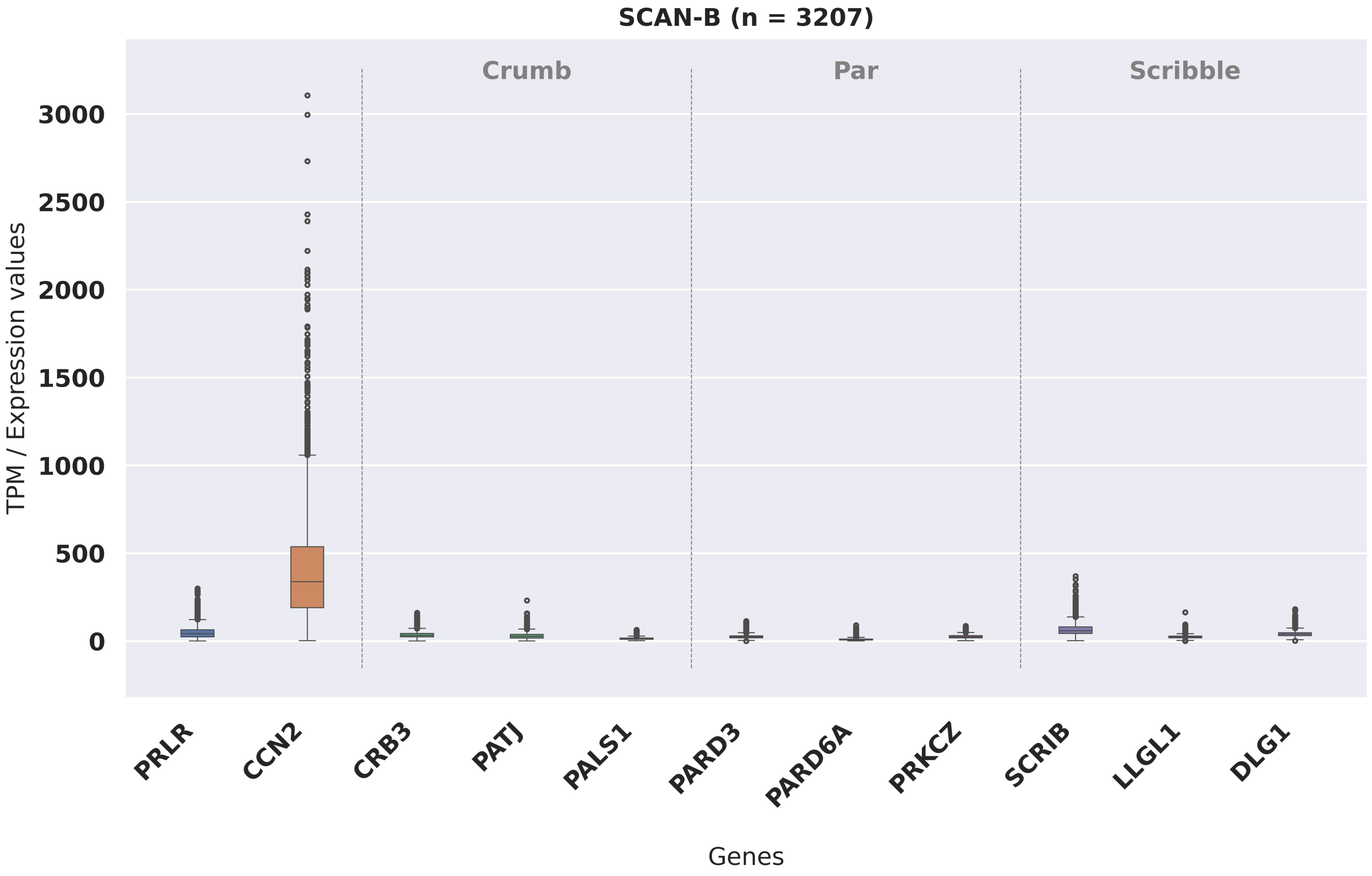

S6. Boxplot comparative view of PRLR and CCN2 gene expressions.

The expression was measured in TPM with outlier values, along with genes associated with Crumb (CRB3, PATJ, PALS1), Par (PARD3, PARD6A, PRKCZ) and Scribble (SCRIB, LLGL1, DLG1) polarity protein complexes in the SCAN-B dataset.

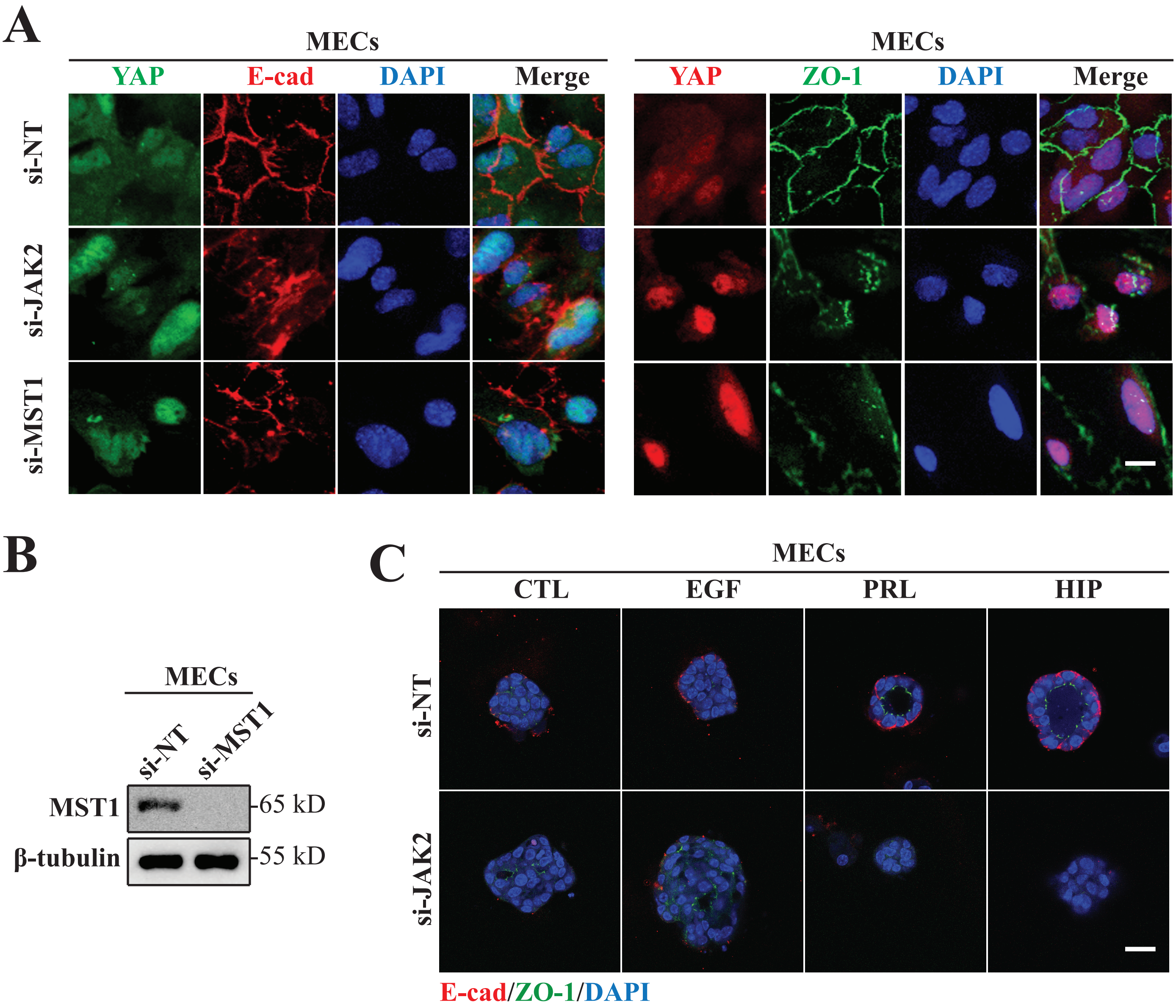

S7. Cell-cell junctions and acini morphogenesis are impaired in JAK2 and MST1 KD MECs.

A. Primary MECs were transfected with either si-NT, si-JAK2 or si-MST1. Cells were then co-immuno-stained with antibodies to YAP (green) and E-cad (red) (left panel) or co-immuno-stained with antibodies to YAP (red) and ZO-1 (green) (right panel). Nuclei were counter stained with DAPI (blue). Scale bar, 5 µm. **B**. Primary MECs were transfected with either si-NT or si-MST1, and the immunoblot analysis of MST1 from total cell lysates were carried out confirming knockdown of MST1. **C**. Control (si-NT) and si-JAK2 cells grown in 3D culture conditions (CTL, EGF, PRL or HIP), and then they were co-stained with antibodies to ZO-1 (green) and E-cad (red). Nuclei were counter stained with DAPI (blue). Scale bar, 20 µm.

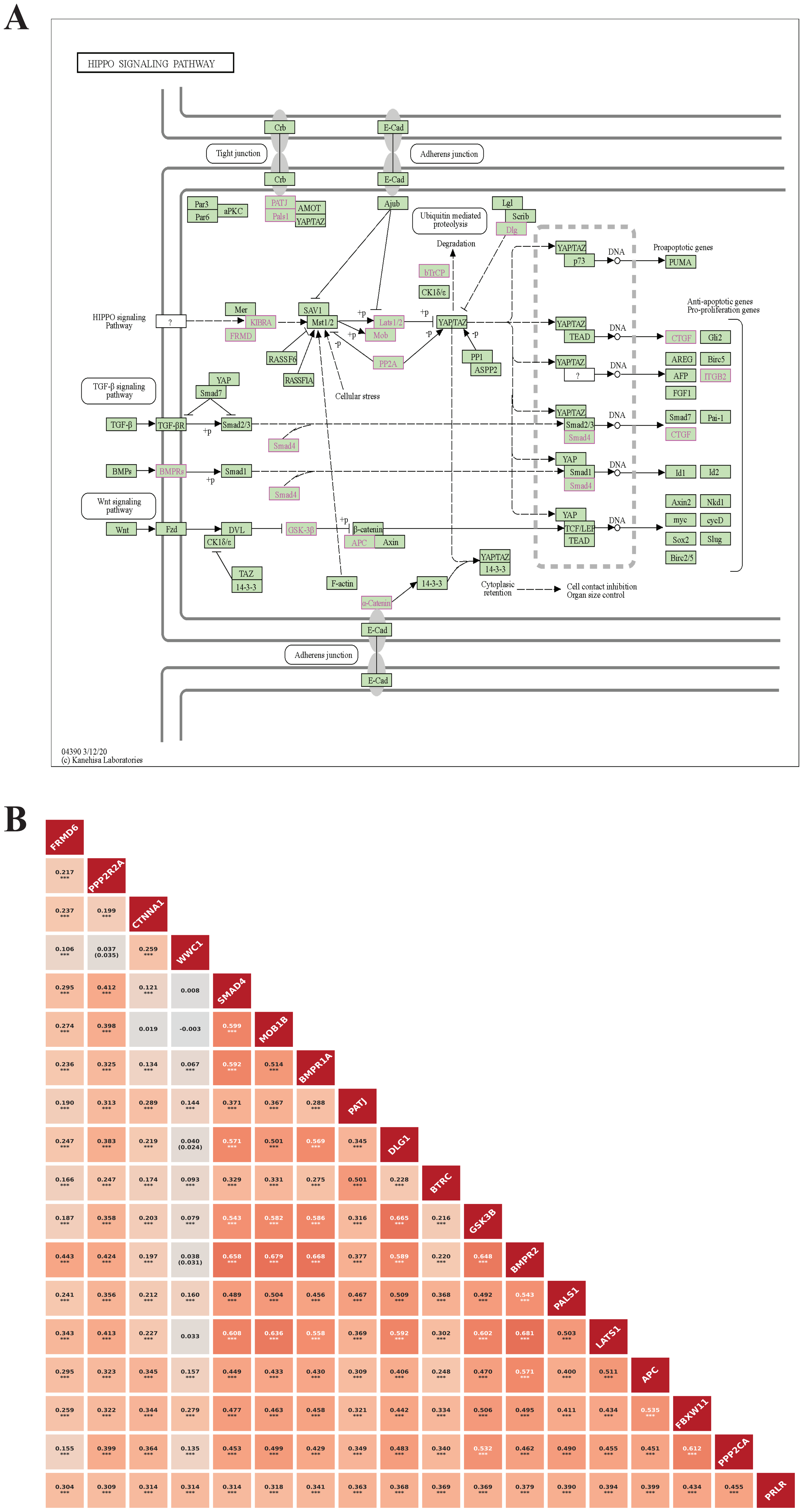

S8. Correlation between PRLR and Hippo signaling pathway and DMFS of PRLR highly related genes.

A. KEGG pathway for the Hippo signaling pathway (hsa04390). Genes highlighted in magenta exhibited a positive co-expression correlation (r > 0.3) with the PRLR gene identified in the SCAN-B dataset. **B.** The heatmap displays the Pearson correlation coefficients between gene expressions of 158 genes belonging to the Hippo pathway (hsa04390) in the SCAN-B dataset. Only correlations with coefficient (r) greater than 0.3 are shown. Each cell in the heatmap represents the correlation coefficient between two genes (pairwise) followed by its q-values (*** q-value < 0.001) in parentheses. Pairs without a q-value are not statistically significant.

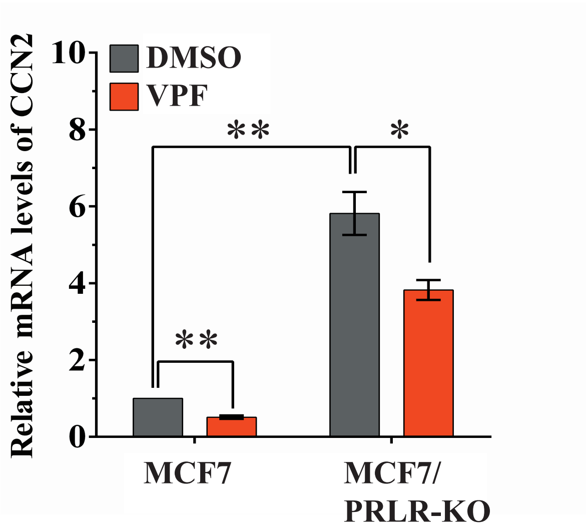

S9. Relative mRNA levels of CCN2 in MCF7 WT and MCF7/PRLR-KO cells with or without VPF treatment for 48 hours.

Graph shows mean ± SEM of triplicates of three independent experiments * $p<0.05$ and ** $p<0.01$ (unpaired two-tailed Student's t-test).

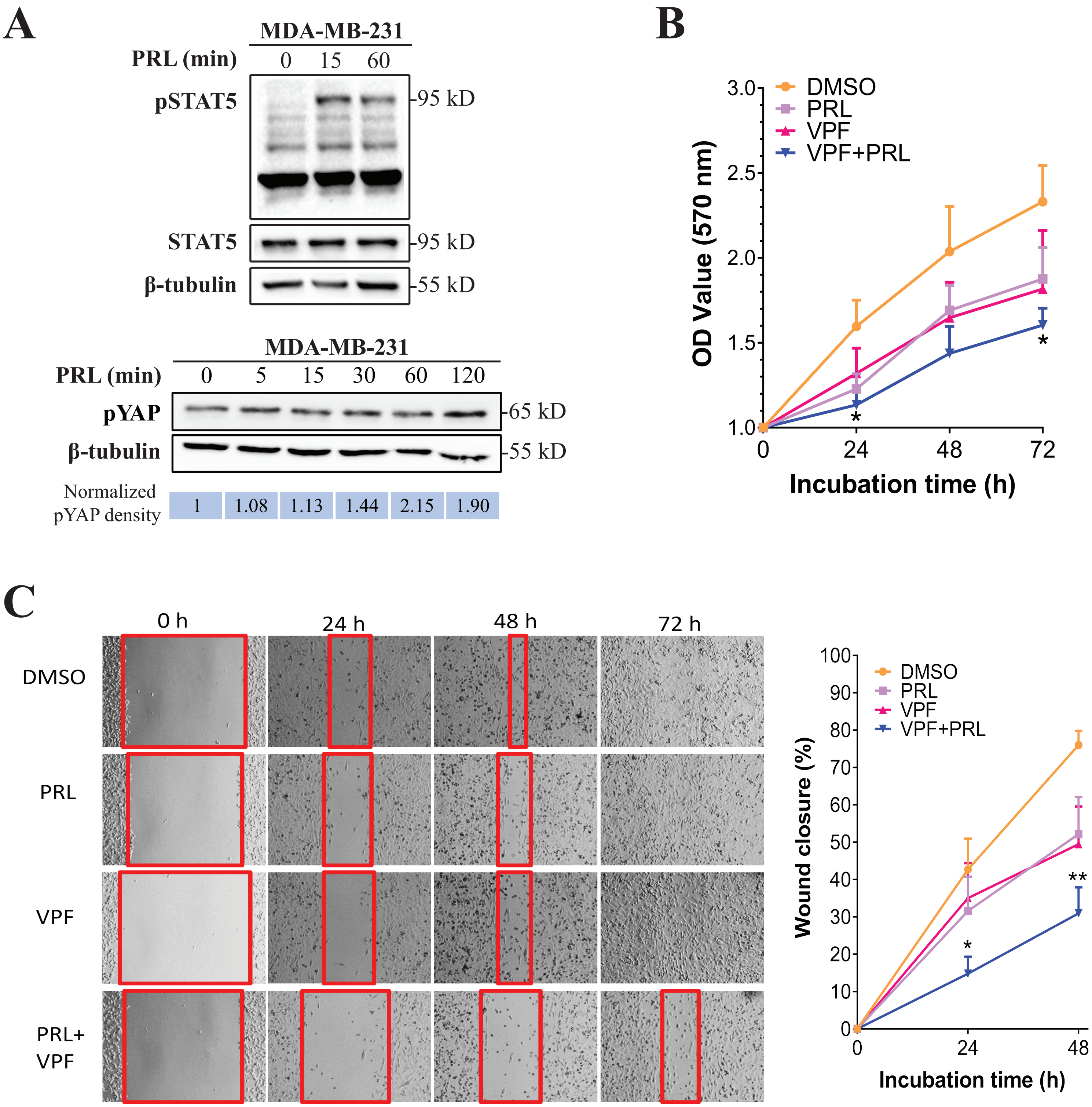

S10. YAP phosphorylation, cell viability (MTT) and cell migration (Wound healing) assays in MDA-MB-231 cells following treatments as indicated in legend.

A. Total cell lysates of MDA-MB-231 cells were immunodetected using antibody to phospho-STAT5 (upper panel) or antibody to pYAP (lower panel). Relative value of pYAP was normalized by β-tubulin. **B**. Cell viability (MTT) assays following treatments with either PRL, VPF or PRL+VPF. Graph showed mean ± SEM of 5 independent experiments. $*p \le 0.05$ (unpaired two-tailed Student's t-test). **C**. Cell migration (Wound healing) assays following treatments with either PRL, VPF or PRL+VPF. Graph showed mean ± SEM of 4 independent experiments. $*p \le 0.05$ and $**p \le 0.001$ (unpaired two-tailed Student's t-test).

Table S1 SCAN-B correlation

Symbol	Pearson correlation (r)
ITGB2	-0.320515558
ACTB	-0.24826381
TGFB1	-0.232518752
WNT6	-0.219224843
TCF7L1	-0.210096402
WNT10A	-0.202334051
CCND3	-0.201633347
TEAD4	-0.199588598
BMP2	-0.196447657
ID1	-0.195347347
WNT2	-0.186223375
TCF7	-0.186009849
BIRC5	-0.156164945
RASSF1	-0.15578736
FZD7	-0.136002809
WNT5B	-0.134795209
FZD9	-0.131398362
WNT10B	-0.125067397
BMP6	-0.119705895
FZD2	-0.119264646
TEAD3	-0.101593514
WNT1	-0.09951933
GDF5	-0.098459889
PPP1CA	-0.095675811
WNT7A	-0.09520093
DVL1	-0.093846565
SERPINE1	-0.092352636
FRMD1	-0.091553709
SCRIB	-0.090239599
WTIP	-0.088793207
FZD5	-0.088181717
GDF7	-0.087764259
LLGL1	-0.084708831
TEAD2	-0.081295186
DVL3	-0.077038763
DVL2	-0.076756545
PPP2R2B	-0.07300566
BIRC3	-0.071718898
CCND2	-0.070168833
BMP7	-0.068439644
SOX2	-0.064933264
CRB2	-0.062688476
FZD10	-0.061597618
FZD1	-0.06043815
WNT16	-0.060067503
BMP8A	-0.058592942
WNT8B	-0.058265234
WNT9B	-0.054481464
BMP8B	-0.053307573

TGFBR2	-0.047902743
AMH	-0.046929357
SNAI2	-0.046729114
WNT3A	-0.045180117
CCN2	-0.043200952
TP53BP2	-0.029101819
PARD6A	-0.027614055
LLGL2	-0.020945123
PPP2R1A	-0.020846678
TCF7L2	-0.018380467
GLI2	-0.018051295
CSNK1E	-0.017745158
CSNK1E	-0.017745158
LATS2	-0.01726447
TP73	-0.016281505
BMP4	-0.01105475
MYC	-0.004434722
APC2	-0.004275771
WWTR1	2.58435E-05
SMAD7	0.003467752
ACTG1	0.005088312
NKD2	0.007595773
BMP5	0.010131001
PARD3	0.010566512
WNT8A	0.021517552
CTNNA2	0.021729613
WNT2B	0.02412613
PARD6G	0.026531112
FZD8	0.030078482
CTNNA3	0.030713186
AMOT	0.034553023
DLG4	0.036119449
GDF6	0.038412883
DLG2	0.042742301
FGF1	0.045135716
AXIN1	0.050964178
LEF1	0.054857534
WNT11	0.056378771
WNT9A	0.059292289
NKD1	0.060375748
PPP2R1B	0.062482478
BBC3	0.071816324
AREG	0.07462901
PRKCZ	0.078285363
YWHAH	0.079074923
FZD4	0.080559472
PPP2R2C	0.081953316
NF2	0.090591067
ID2	0.09441805
AXIN2	0.097094745
AFP	0.098436619

YWHAQ	0.1014224
CRB1	0.103600075
PPP2R2D	0.105298737
YWHAG	0.107014111
CSNK1D	0.107668201
SMAD3	0.119560532
TGFB2	0.12597428
AJUBA	0.126173477
CTNNB1	0.126392267
WNT7B	0.129344903
CCND1	0.13492256
YWHAZ	0.138535863
FZD6	0.139324063
WNT3	0.14207239
CDH1	0.14678507
BMPR1B	0.149434739
YWHAE	0.15124896
YAP1	0.15291656
WNT5A	0.154074157
SMAD2	0.168702576
BIRC2	0.178869798
WNT4	0.181721293
SAV1	0.185571459
PPP1CB	0.187618294
FZD3	0.188641834
DLG5	0.190950002
RASSF6	0.20892178
PRKCI	0.210743978
TEAD1	0.212807275
TGFB3	0.215103279
SMAD1	0.219528432
TGFBR1	0.220102102
MOB1A	0.224830722
YWHAB	0.238746814
PARD6B	0.242671871
DLG3	0.244132201
LIMD1	0.249766028
PPP2CB	0.25735768
PPP1CC	0.259345126
STK3	0.269660875
FRMD6	0.303731099
PPP2R2A	0.30888987
CTNNA1	0.313935321
WWC1	0.314104013
SMAD4	0.31413287
MOB1B	0.317972035
BMPR1A	0.340800367
PATJ	0.363374378
DLG1	0.367726857
BTRC	0.368611833
GSK3B	0.36889643

BMPR2	0.378583672
PALS1	0.389952285
LATS1	0.394018581
APC	0.399027138
FBXW11	0.434151119
PPP2CA	0.455362945
PRLR	1

Table S2 TCGA Cancer types

Study Abbreviation	Study Name	Samples
LAML	Acute Myeloid Leukemia	126
ACC	Adrenocortical carcinoma	79
BLCA	Bladder Urothelial Carcinoma	414
LGG	Brain Lower Grade Glioma	532
BRCA	Breast invasive carcinoma	1134
CESC	Cervical squamous cell carcinoma and endocervical adenocarcinoma	306
CHOL	Cholangiocarcinoma	36
COAD	Colon adenocarcinoma	505
ESCA	Esophageal carcinoma	185
GBM	Glioblastoma multiforme	170
HNSC	Head and Neck squamous cell carcinoma	504
KICH	Kidney Chromophobe	66
KIRC	Kidney renal clear cell carcinoma	544
KIRP	Kidney renal papillary cell carcinoma	291
LIHC	Liver hepatocellular carcinoma	374
LUAD	Lung adenocarcinoma	542
LUSC	Lung squamous cell carcinoma	504
DLBC	Lymphoid Neoplasm Diffuse Large B-cell Lymphoma	48
MESO	Mesothelioma	87
OV	Ovarian serous cystadenocarcinoma	430
PAAD	Pancreatic adenocarcinoma	179
PCPG	Pheochromocytoma and Paraganglioma	184
PRAD	Prostate adenocarcinoma	506
READ	Rectum adenocarcinoma	167
SARC	Sarcoma	263
SKCM	Skin Cutaneous Melanoma	472
STAD	Stomach adenocarcinoma	416
TGCT	Testicular Germ Cell Tumors	156
THYM	Thymoma	120
THCA	Thyroid carcinoma	513
UCS	Uterine Carcinosarcoma	57
UCEC	Uterine Corpus Endometrial Carcinoma	554
UVM	Uveal Melanoma	80
	TOTAL	10544

Table S3 OS and RFS_results

OS

Symbol	Probe_ID	Order	Patients	HR (CI)	logrank P	Low	High	Lifespan
FRMD6	225481_at	1						
PPP2R2A	228013_at	2	943	0.87 (0.66 – 1.13)	0.292	75.83	89.04	13.21
CTNNA1	200765_x_at	3	943	1.05 (0.80 – 1.37)	0.713	90.00	79.20	-10.80
WWC1	213085_s_at	4	943	1.02 (0.78 – 1.33)	0.899	85.20	84.00	-1.20
SMAD4	235725_at	5	943	0.98 (0.75 – 1.28)	0.894	81.87	84.00	2.13
MOB1B	225997_at	6	943	0.97 (0.74 – 1.27)	0.827	81.60	89.03	7.43
BMPR1A	213578_at	7	943	1.02 (0.78 – 1.33)	0.906	81.87	85.20	3.33
PATJ	214493_s_at	8	943	1.05 (0.80 – 1.37)	0.735	90.00	84.00	-6.00
DLG1	202515_at	9	943	0.93 (0.71 – 1.21)	0.570	79.20	90.00	10.80
BTRC	224471_s_at	10	943	0.88 (0.67 – 1.15)	0.341	75.83	90.00	14.17
GSK3B	226191_at	11	943	0.83 (0.64 – 1.09)	0.176	75.83	90.00	14.17
BMPR2	225144_at	12	943	0.79 (0.60 – 1.03)	0.083	69.73	99.60	29.87
PALS1	219321_at	13	943	0.78 (0.60 – 1.02)	0.069	69.60	99.60	30.00
LATS1	227772_at	14	943	0.80 (0.61 – 1.05)	0.112	70.68	99.60	28.92
APC	203527_s_at	15	943	0.80 (0.61 – 1.05)	0.108	70.68	99.60	28.92
FBXW11	209455_at	16	943	0.78 (0.59 – 1.02)	0.064	69.73	106.80	37.07
PPP2CA	208652_at	17	943	0.84 (0.64 – 1.10)	0.205	75.83	99.60	23.77
PRLR	227629_at	18	943	0.74 (0.57 – 0.97)	0.031	67.40	108.00	40.60

RFS

Symbol	Probe_ID	Order	Patients	HR (CI)	logrank P	Low	High	Relapse
FRMD6	225481_at	1						
PPP2R2A	228013_at	2	2032	0.79 (0.68 – 0.92)	0.002400	32.03	44.00	11.97
CTNNA1	200765_x_at	3	2032	0.85 (0.73 – 0.99)	0.035900	34.00	43.00	9.00
WWC1	213085_s_at	4	2032	0.87 (0.74 – 1.01)	0.059600	34.00	41.04	7.04
SMAD4	235725_at	5	2032	0.80 (0.69 – 0.93)	0.003900	32.60	43.70	11.10
MOB1B	225997_at	6	2032	0.81 (0.70 – 0.95)	0.007400	33.00	42.05	9.05
BMPR1A	213578_at	7	2032	0.83 (0.71 – 0.96)	0.014200	34.00	42.00	8.00
PATJ	214493_s_at	8	2032	0.82 (0.71 – 0.96)	0.010800	32.82	43.00	10.18
DLG1	202515_at	9	2032	0.81 (0.70 – 0.94)	0.005900	34.04	43.00	8.96
BTRC	224471_s_at	10	2032	0.73 (0.63 – 0.85)	0.000044	30.00	45.08	15.08
GSK3B	226191_at	11	2032	0.72 (0.62 – 0.84)	0.000018	30.00	45.44	15.44
BMPR2	225144_at	12	2032	0.79 (0.68 – 0.92)	0.002200	33.00	43.93	10.93
PALS1	219321_at	13	2032	0.78 (0.67 – 0.90)	0.001000	33.00	44.40	11.40
LATS1	227772_at	14	2032	0.76 (0.65 – 0.88)	0.000300	33.00	44.40	11.40
APC	203527_s_at	15	2032	0.75 (0.64 – 0.87)	0.000200	32.82	45.00	12.18
FBXW11	209455_at	16	2032	0.72 (0.62 – 0.84)	0.000018	31.51	45.60	14.09
PPP2CA	208652_at	17	2032	0.78 (0.67 – 0.91)	0.001200	34.00	43.93	9.93
PRLR	227629_at	18	2032	0.74 (0.64 – 0.86)	0.000025	30.00	46.80	16.80

Table S4 List of antibodies used in Western blot and IF staining

Antibodies/Reagents	Vendor Cat#	Application
**Primary Antibodies**		
anti-E-Cad rat monoclonal antibody	Sigma #U3254	1:200 IF
anti-ZO-1 mouse monoclonal antibody,Alexa Fluor® 488	Invitrogen #339188	1:100 IF
anti-β-casein mouse monoclonal antibody	Santa-Cruz #sc-166520	1:100 IF
anti-Par6 rabbit polyclonal antibody	abcam #ab49776	1:100 IF
anti-PKCζ rabbit polyclonal antibody	abcam #ab108970	1:100 IF
anti-Par3 rabbit polyclonal antibody	Millipore #07-330	1:100 IF
anti-Crb3 rat monoclonal antibody	abcam #ab180835	1:100 IF
anti-Hugl-1 goat polyclonal antibody	Santa-Cruz #sc-49990	1:100 IF
anti-phospho-YAP rabbit monoclonal antibody	Cell Signaling #13008	1:1000 WB
anti-YAP rabbit polyclonal antibody	Cell Signaling #4912	1:1000 WB, 1:100 IF
anti-phospho-Stat5 rabbit polyclonal antibody	Invitrogen #71-6900	1:1000 WB
anti-Stat5a mouse monoclonal antibody	ThermoFisher #13-3600	1:1000 WB
anti-β-Tubulin mouse monoclonal antibody	Santa-Cruz #sc-53140	1:10000 WB
anti-PRLR mouse monoclonal antibody	Santa-Cruz #sc-377098	1:1000 WB
anti-phospho-MST1 rabbit monoclonal antibody	Cell Signaling #49332	1:1000 WB
anti-MST1 rabbit polyclonal antibody	Cell Signaling #3682	1:1000 WB
anti-Jak2 mouse monoclonal antibody	Santa-Cruz #sc-390539	1:1000 WB
anti-CK18 mouse monoclonal antibody	Santa-Cruz #sc-32329	1:100 IF
anti-ERα mouse monoclonal antibody	Santa-Cruz #sc-8002	1:100 IF
anti-CD44 mouse monoclonal antibody	Santa-Cruz #sc-7297	1:100 IF
anti-Vimentin rabbit monoclonal antibody	abcam #ab92547	1:200 IF
anti-PCNA Rabbit monoclonal antibody	Cell Signaling #13110	1:1000 WB
anti-Ki67 rabbit monoclonal antibody	abcam #ab16667	1:200 IF
**Secondary Antibodies**		
goat anti-rabbit IgG HRP	Santa-Cruz #sc-2004	1:5000 WB
goat anti-mouse IgG-HRP	Santa-Cruz #sc-2005	1:5000 WB
donkey anti-rabbit IgG (H+L) Fluor 488	Invitrogen #A21206	1:100 IF
donkey anti-rabbit IgG (H+L) Fluor 546	Invitrogen #A10040	1:100 IF
donkey anti-mouse IgG (H+L) Fluor 488	Invitrogen #A21202	1:100 IF
goat anti-mouse IgG (H+L) Fluor 568	Invitrogen #A11004	1:100 IF
goat anti-rat IgG (H+L) Fluor 488	Invitrogen #A11006	1:100 IF
goat anti-rat IgG (H+L) Fluor 555	Invitrogen #A21434	1:100 IF
donkey anti-goat IgG-R Rhodamine conjugated	Santa-Cruz #sc-2094	1:100 IF

Figure 4B

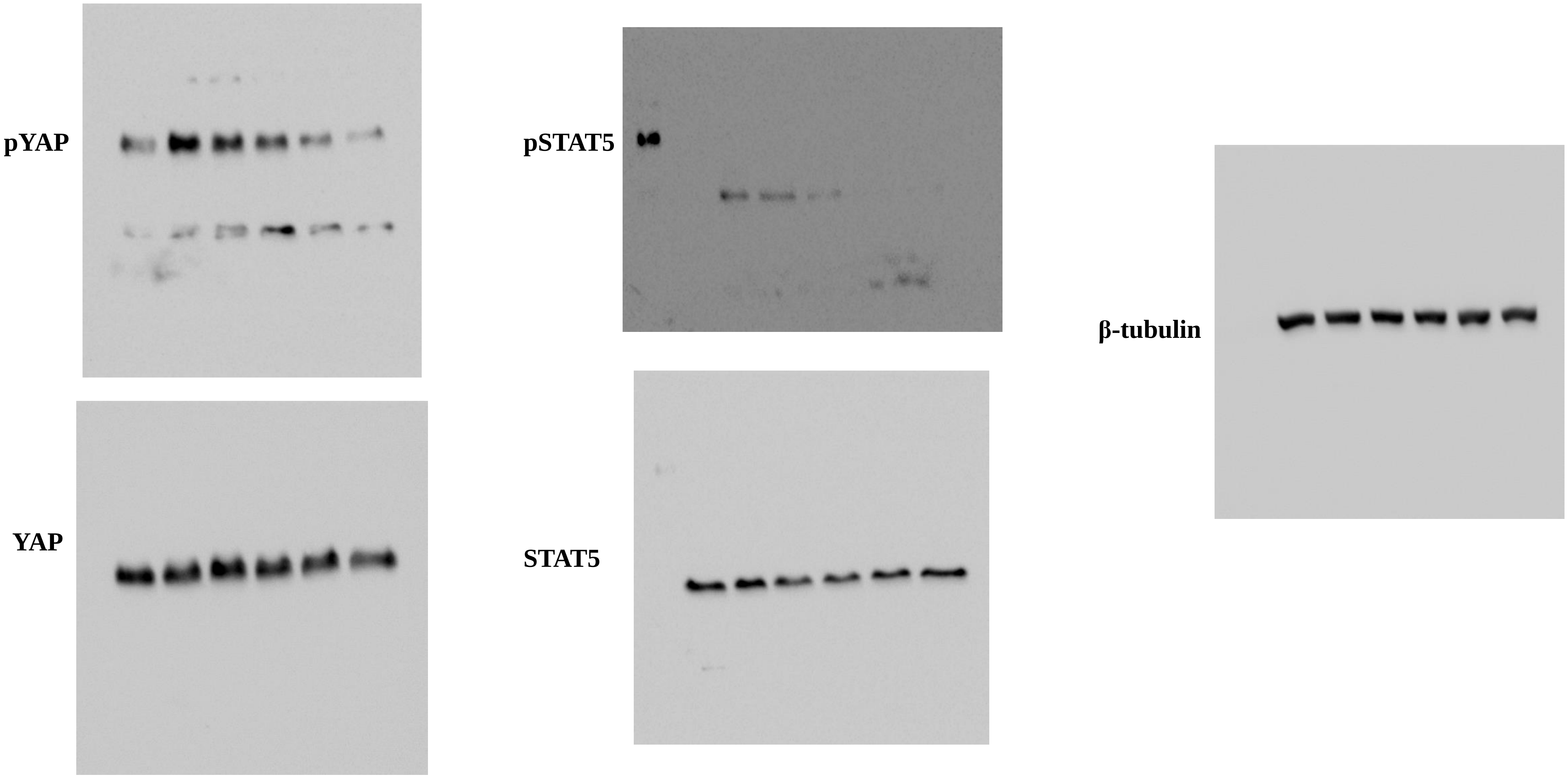

Figure 4D

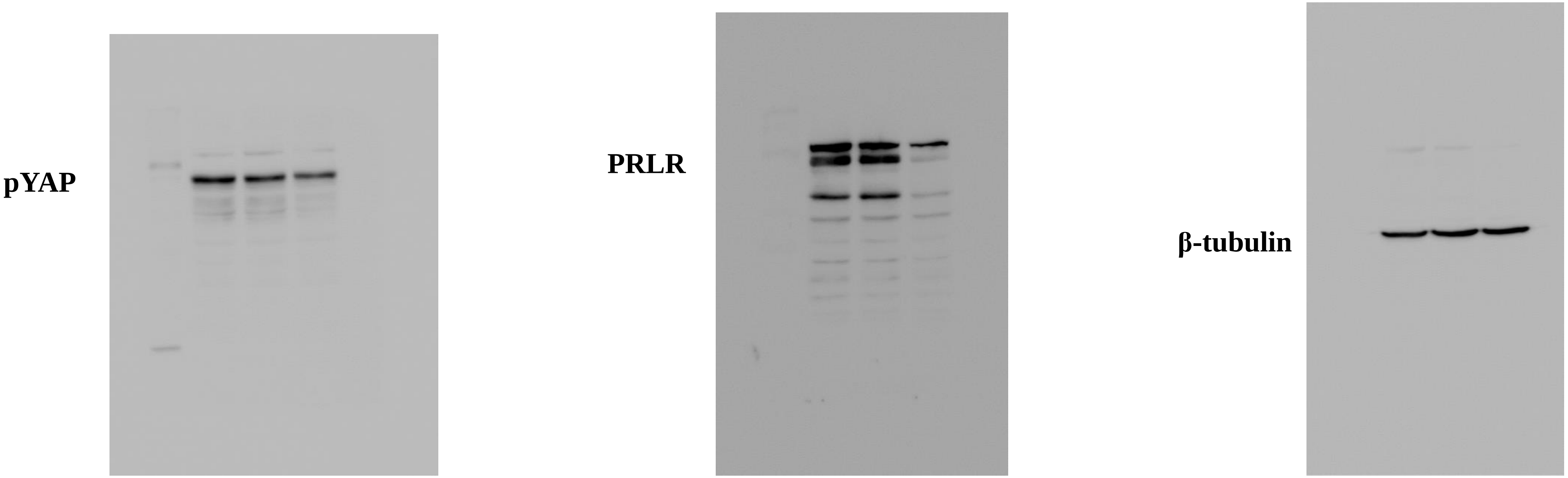

Figure 5A

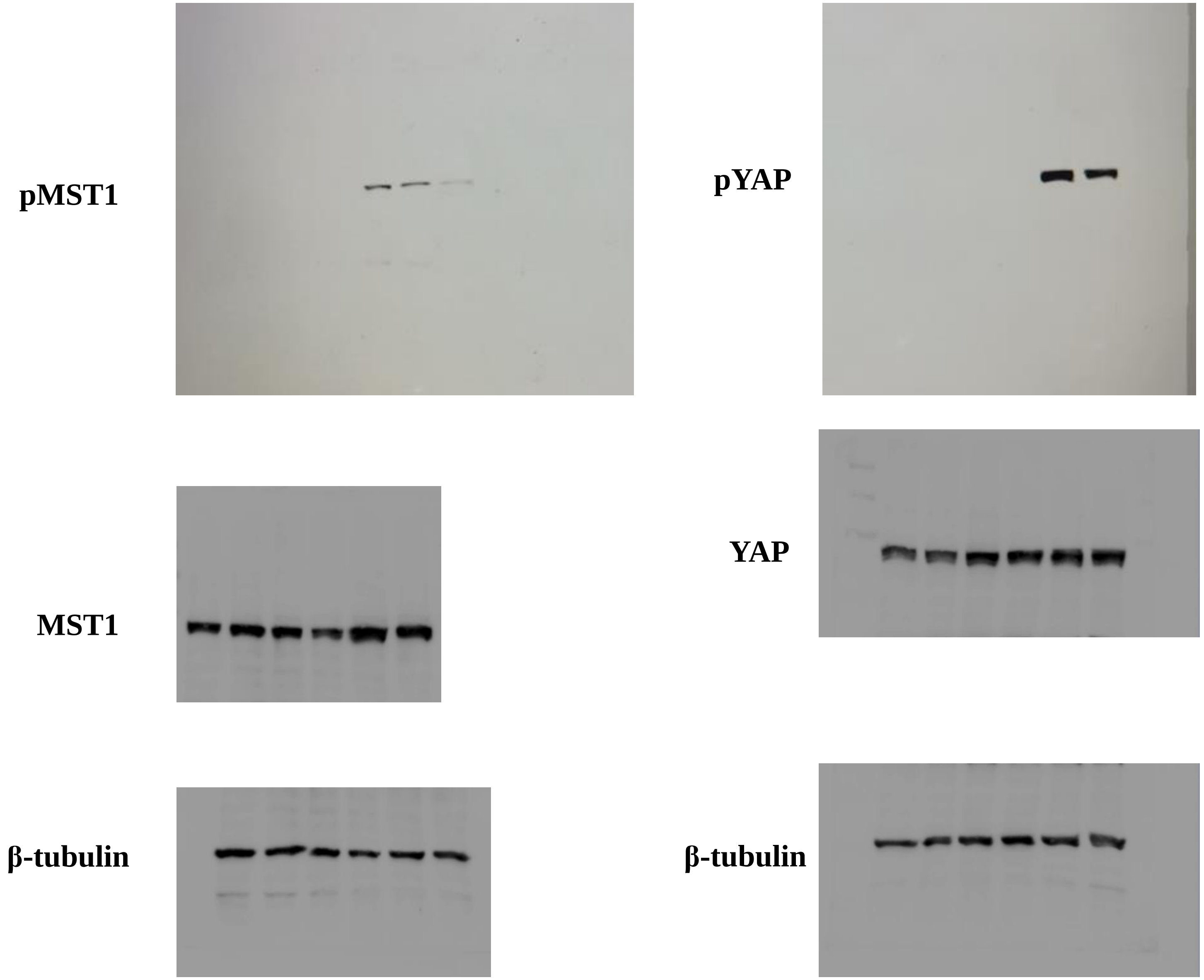

Figure 5C

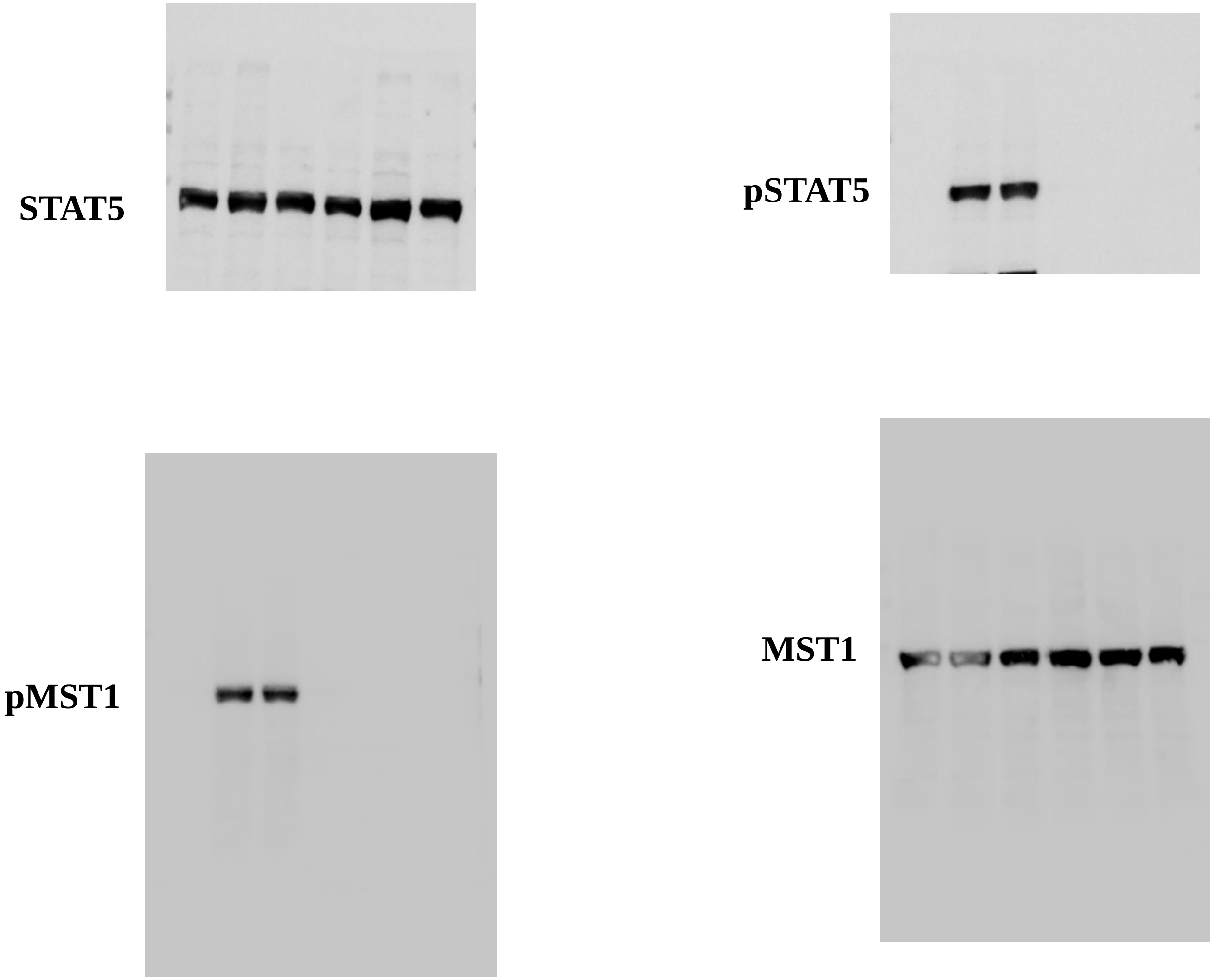

Figure 8E

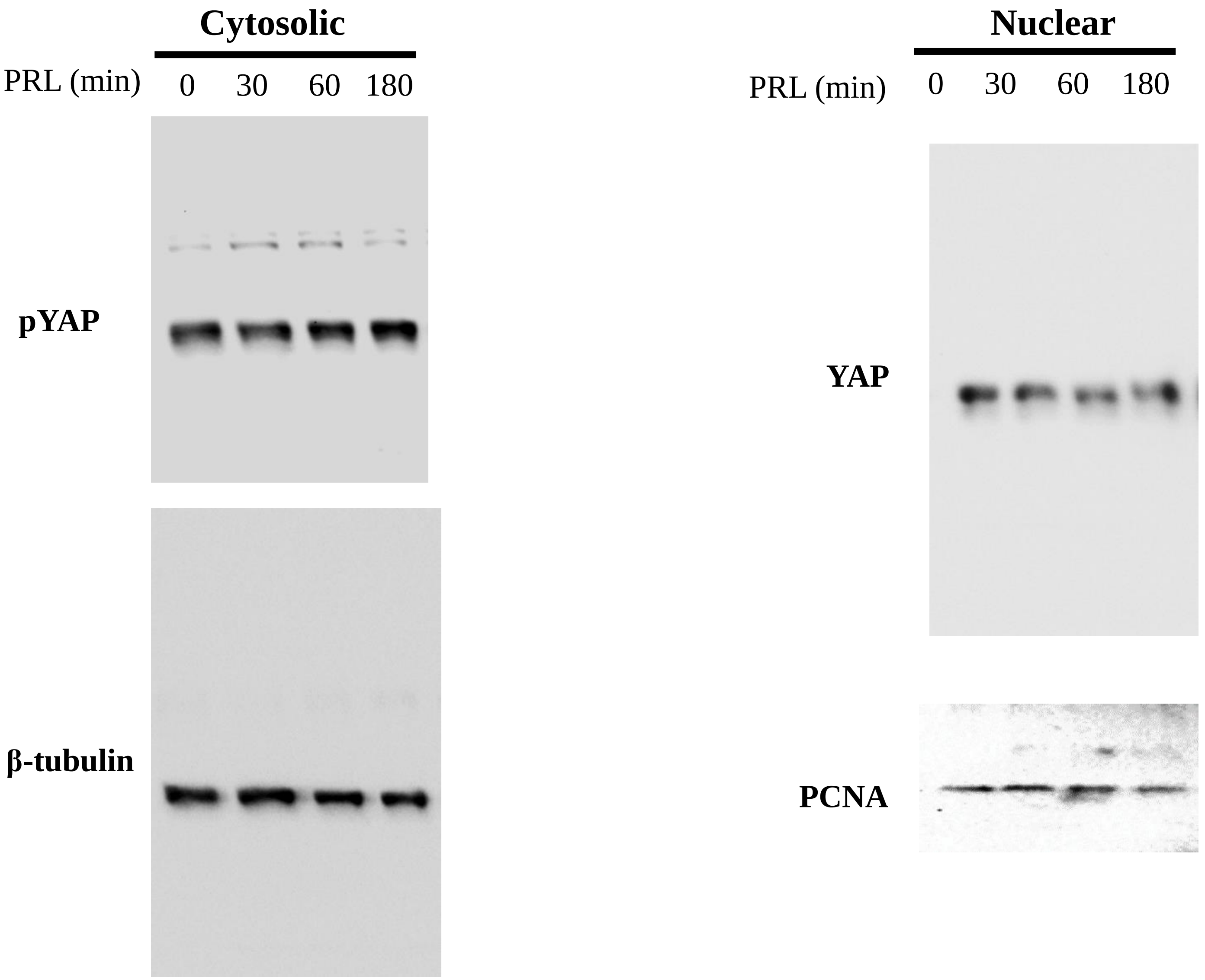
Cytosolic
PRL (min) 0 30 60 180
pYAP
β-tubulin
Nuclear
PRL (min) 0 30 60 180
YAP
PCNA
